# Astragaloside IV alleviates 1-deoxysphinganine-induced mitochondrial dysfunction during the progression of chronic kidney disease through p62-Nrf2 antioxidant pathway

**DOI:** 10.3389/fphar.2023.1092475

**Published:** 2023-03-24

**Authors:** Ting Gui, Qingfa Chen, Jiangsong Li, Ke Lu, Chen Li, Bin Xu, Yang Chen, Jingwen Men, Gerd A. Kullak-Ublick, Weihua Wang, Zhibo Gai

**Affiliations:** ^1^ Innovation Research Institute of Traditional Chinese Medicine, Shandong University of Traditional Chinese Medicine, Jinan, China; ^2^ Research Center of Basic Medicine, Jinan Central Hospital, Jinan, China; ^3^ Institute for Tissue Engineering and Regenerative Medicine, Liaocheng University/Liaocheng People’s Hospital, Liaocheng, China; ^4^ Department of Urology, Liaocheng People’s Hospital, Liaocheng, China; ^5^ College of Traditional Chinese Medicine, Shandong University of Traditional Chinese Medicine, Jinan, China; ^6^ Department of Clinical Pharmacology and Toxicology, University Hospital Zurich, University of Zurich, Zurich, Switzerland; ^7^ Mechanistic Safety, CMO and Patient Safety, Global Drug Development, Novartis Pharma, Basel, Switzerland; ^8^ The Central Laboratory, Liaocheng People’s Hospital, Liaocheng, China; ^9^ Experimental Center, Shandong University of Traditional Chinese Medicine, Jinan, China; ^10^ Key Laboratory of Traditional Chinese Medicine Classical Theory, Ministry of Education, Shandong University of Traditional Chinese Medicine, Jinan, China; ^11^ Shandong Provincial Key Laboratory of Traditional Chinese Medicine for Basic research, Shandong University of Traditional Chinese Medicine, Jinan, China

**Keywords:** chronic kidney disease, 1-deoxysphingolipids, mitochondrial dysfunction, astragaloside IV, nuclear factor erythroid-related factor 2, p62

## Abstract

**Introduction:** Chronic kidney disease (CKD) can lead to significant elevation of 1-deoxysphingolipids (1-deoxySL). The increase of 1-deoxySL in turn can result in mitochondrial damage and oxidative stress, which can cause further progression of CKD.

**Methods:** This study assessed the therapeutic effect of Astragaloside IV (AST) against 1-deoxySL-induced cytotoxicity *in vitro* and in rats with CKD. HK-2 cells were exposed to 1-deoxysphinganine (doxSA) or doxSA + AST. doxSA-induced mitochondrial dysfunction and oxidative stress were evaluated by immunostaining, real-time PCR, oxidative stress sensor, and transmission electron microscopy. The potential effects of AST on kidney damage were evaluated in a rat 5/6 nephrectomy (5/6 Nx) model of CKD.

**Results:** The findings of *in vitro* experiments showed that doxSA induced mitochondrial damage, oxidative stress, and apoptosis. AST markedly reduced the level of mitochondrial reactive oxygen species, lowered apoptosis, and improved mitochondrial function. In addition, exposure to AST significantly induced the phosphorylation of p62 and the nuclear translocation of Nrf2 as well as its downstream anti-oxidant genes. p62 knock-down fully abolished Nrf2 nuclear translocation in cells after AST treatment. However, p62 knock-down did not affect TBHQ-induced Nrf2 nuclear translocation, indicating that AST can ameliorate doxSA-induced oxidative stress through modulation of p62 phosphorylation and Nrf2 nuclear translocation.

**Conclusion:** The findings indicate that AST can activate Nrf2 antioxidant pathway in a p62 dependent manner. The anti-oxidative stress effect and the further mitochondrial protective effect of AST represent a promising therapeutic strategy for the progression of CKD.

## Introduction

As an essential public health problem, chronic kidney disease (CKD) can affect up to 13% of adults in America (Coresh et al., 2007) and worldwide (Ruiz-Ortega et al., 2020). In the progression of CKD, oxidative stress plays a key role in promoting kidney damage and the subsequent nephron loss, decline in renal function, and ultimately end-stage kidney disease (ESKD) (Irazabal and Torres, 2020). Oxidative stress is commonly observed in several renal disorders through the elevation of intracellular levels of reactive oxygen species (ROS) and/or reactive nitrogen species (RNS) (Massy et al., 2009; Putri and Thaha, 2014; Irazabal and Torres, 2020). Although, researchers have shown elevated levels of ROS in patients with CKD and animal models of kidney injury, the underlying sources of increased ROS and the signaling mechanisms involved, leading to kidney damage, remain far less known yet.

Astragalus membranaceus, also known as Huangqi, is a traditional Chinese medicine widely used for the management of kidney diseases (Zhang et al., 2019). A large number of clinical observations have demonstrated that Astragalus is safe for long-term use (Lin et al., 2019). Astragaloside IV (AST), a saponin extract of the Astragalus root, is one of the main active ingredients of Astragalus. In recent years, few studies have indicated that AST can exhibit significant renal protective effects and can effectively attenuate renal fibrosis (Guo et al., 2016; Wang et al., 2020). In addition, AST has been reported to attenuate cisplatin-induced acute kidney injury (Yan et al., 2017; Qu et al., 2020) and drug-associated chronic nephropathy (Gao et al., 2020).

Anti-oxidative stress is one of the primary mechanisms to facilitate the renal protection activity of AST (Zhang et al., 2020). Nuclear factor erythroid-related factor 2 (Nrf2) is the primary defense mechanism against oxidative stress, which can regulate the transcription of >300 antioxidant response element-regulated genes (Zhang and Chapman, 2020). Moreover, a prior report has suggested that AST could protect renal cells from oxidative stress-induced injury by activating Nrf2 (Chen et al., 2018a; Wang et al., 2019).

We have previously reported that intracellular oxidative stress was related to the increase of 1-deoxysphingolipids in CKD under both *in vivo* and *in vitro* conditions (Gui et al., 2021). Abnormal 1-deoxysphingolipid levels have been found to exert substantial cytotoxic effects, while the underlying mechanisms of 1-deoxysphingolipids and related cellular toxic processes remain obscure. In this study, we have performed an untargeted metabolomics analysis and a series of functional tests to reveal that 1-deoxysphinganine (doxSA) could induce mitochondrial ROS production following mitochondrial damage and inhibition of mitochondrial energy production. Such mitochondrial impairments were ameliorated by AST treatment through affecting the p62-NRF2 antioxidant pathway under *in vitro* and *in vivo* settings.

## Material and methods

### Animals

Twelve male Sprague-Dawley rats (200–220 g per rat) were purchased from Vital River Laboratories (Beijing, China). In the animal facility, rats were kept in the separate cage with three rats per cage and were subjected to 12:12-h day-night cycle. Animals had *ad libitum* access to water and conventional chow (AIN93 M, Keao Xieli feed co. LTD., Beijing, China). Rats were randomly divided into a 5/6 nephrectomy (5/6 Nx) group and a normal control sham-operated group (6 rats per group). Lipidomics analysis was performed using five animals per group. In another experiment, lipidomics results were confirmed and further analyzed by using at least six animals per group. Astragaloside IV was suspended in 1% carboxymethyl cellulose (CMC) solution. Astragaloside IV group mice received astragaloside IV treatment by oral administration at a dosage of 10 mg/kg daily for 4 weeks. At the end of the study, all mice were anesthetized with an intraperitoneal injection of 2.0% pentobarbital sodium and then sacrificed. At the end of the study, all the rats were sacrificed under anesthesia and kidneys were harvested 4 weeks after the second stage surgery.

### Cell culture

HK-2 (human kidney-2) cells were cultured in K-SFM medium (17,005-042, GIBCO), which was supplemented with the bovine pituitary extract (0.05 mg/ml) as well as human recombinant epidermal growth factor (5 ng/ml), and incubated at 37°C in 5% CO2 incubator. HK2 cells were treated with 200 μM AS-IV and/or 5 μM doxSA for 24 h. For siRNA transfection experiment, HK2 cells were treated with 200 μM AST or 25 μM TBHQ for 24 h and harvested 72 h after siRNA transfection.

### Lipidomics analysis

The lipidomic analysis was performed as described previously (Pellegrino et al., 2014). Linearity R2>0.9 and CV<20% were selected as the quality criteria for the identified lipid metabolites. Analysis of the sphingoid base profile was performed as described earlier, through lipids acid/base hydrolyzation to remove the N-linked fatty acid and headgroups. Briefly, acid/base–hydrolyzed lipids were derivatized with ο-phthalaldehyde (OPA) by redissolving in 75 μL of 56.7% MeOH, 33.3% EtOH, 10% H2O and 5 μL OPA working solution (990 μL boric acid [3%] + 10 μL OPA [50 mg/ml in EtOH] + 0.5 μL 2-mercaptoethanol). Thereafter, the separation of the deoxysphingoid and sphingoid base backbones was carried out by using liquid chromatography coupling a C18 column (Uptispere 120 Å, 5 μm, 125 × 2 mm, Interchim, Montluçon, France) to a Transcend UPLC pump (Thermo, Reinach, BL, Switzerland). The deoxysphingoid and sphingoid bases were then detected using a Q-Exactive hybrid quadrupole Orbitrap mass spectrometer (Thermo, Reinach, BL, Switzerland) run in full scan mode after ionization with an atmospheric pressure chemical ionization source (APCI). The MS parameters used were as follows: mass resolution of 140,000, scan range of m/z 120–1200, max injection time of 512 ms and automatic gain control (ACG) target of 3.00E+06.

### Untargeted metabolomics of isolated mitochondria

The untargeted metabolomic analysis was performed as described previously (Krajnc et al., 2020). Briefly, HK-2 cells were seeded at the density of 1 × 10^6^ cells per dish on 10-cm dishes and were treated for 24 h with 1-deoxysphinganine at 5 μM or an equal volume of EtOH. The cells were then washed with ice-cold PBS and the mitochondria were harvested with isolation buffer (200 mM mannitol, 50 mM sucrose, 5 mM KH2PO4, 5 mM MOPS, 0.1% fatty acid free BSA, 1 mM EGTA, pH 7.5). The isolated mitochondria were treated with prewarmed (70°C) 80% (v/v) methanol and thereafter incubated on ice for 15 min, followed by centrifugation at 15,000 g for 3 min at 4°C. The supernatant was collected and resolved by hydrophilic interaction liquid chromatography in negative mode using a Q-Exactive Hybrid Quadrupole-Orbitrap Mass Spectrometer (ThermoFischer Scientific, Waltham, MA). The metabolite data sets were processed by Progenesis QI (Non-linear Dynamics). For all the samples, relative quantification of the detected compounds was calculated. The detected ions were further identified based on accurate mass, detected adduct patterns, and isotope patterns by comparison with the metabolites listed in the Human Metabolome Database (Wishart et al., 2018). A reference mixture of 29 selected compounds (amino acids, nucleotides, and metabolic intermediates) was analyzed along with the samples for quality control assessment. The coefficients of variation for the biological and technical replicates of all the compounds were near or below 20%. A statistical analysis based on “Between Group” analysis was made with R, using the Bioconductor package maintainer (Culhane et al., 2005).

### Renal pathology assessment and immunostaining

For the histological evaluation, kidney tissues were extracted, sectioned, and stained with standard protocols. Masson’s trichrome staining was performed with Masson Trichrome staining (Sigma-Aldrich, St. Louis, MO, USA) kits following the manufacturers’ instruction. The primary antibody used for immunostaining was anti-alpha smooth muscle actin (SMA) (NBP1-30894, Novus Biologicals, Littleton, CO, USA) immunohistochemical staining was performed with Envision DAB + kits (Dako, Basel, Switzerland) following the manufacturer’s instructions.

### Immunocytochemistry

AST and/or doxSA treated HK-2 cells were fixed in 4% paraformaldehyde in PBS, blocked with 1% BSA/PBS for 30 min, and thoroughly treated with 0.1% Triton X-100 for 15 min. HK2 cells were then incubated with anti-Tom20 and anti-Cleaved Caspase three antibodies overnight at 4°C. The cells were washed three times with PBS and incubated with the secondary antibodies for 1 h in the dark. After washing with PBS, the nuclei was fixed with DAPI (Vector Laboratories) and observed under a fluorescence microscope (Leica DMI6000B). TUNEL kit (ApopTag, Millipore) was used to perform TUNEL experiments. Five fields were randomly selected in the high-power field to count the number of TUNEL-positive cells.

### Estimation of ROS

For intracellular reactive oxygen species (ROS) assays, 80%–90% confluent cells were treated for 24 h according to the manufacturer’s protocol (CellROX; Life Technologies). The subcellular localization of ROS was performed as previously described using a plasmid encoding redox-sensitive GFP (roGFP) from AddGene (plasmid 49,437) to the mitochondrial matrix (Mito-roGFP) (Waypa et al., 2010). Briefly, the fluorescence signals were imaged by confocal microscopy by fluorescence emission at 525 nm after excitation with 405 and 488 nm, separately, or in a plate reader (GloMax Discover; Promega, Madison, WI) by sequential detection at 405 and 475 nm, as well as fuorescence measurement using a 500–550 nm fliter. The fluorescence ratio of the confocal images was calculated after excitation at 405 and 488 nm using ImageJ. The data has been presented as ratios of fluorescence after excitation at 405–488 nm, where high ratios indicate sensor oxidation and low ratios indicate sensor reduction. All the cell culture experiments were performed thrice independently with three technical replicates for each measurement and treatment.

ROS detection in kidney was performed as previously described (Gai et al., 2017). Briefly, fresh renal cortical tissue sections were cut with a razor blade and placed in Hanks solution (10 mM Hepes-NaOH, pH 7.4) with fluorescent ROS probes. The sections were then incubated with 10 μM 2′,7′-dichlorodihydrofluorescein diacetate (2′,7′-DCFDA, Molecular Probes, Inc., Eugene, Oregon, USA) for 10 min at the room temperature, and then washed in Hanks solution for 5 min. A series of threshold images were processed using ImageJ software (National Institutes of Health, Bethesda, MD, USA) to determine the relative intensities of 2′,7′-dichlorofluorescein (2′,7′-DCF).

### RNA isolation and real-time quantitative polymerase chain reaction (qPCR) assays

RNA isolation and RT-qPCR were performed according to the previously described protocol. HK2 cells were washed twice in PBS at 6, 12, 18 and 24 h after AST and/or doxSA treatment. Thereafter, RNA from treated cells and tissues was extracted using Trizol (Invitrogen, CA, USA) according to the manufacturer’s instructions. Then RT and qPCR were performed according to the previously described protocol. Briefly, reverse transcription of 2 μg of RNA was performed by treating with DNAse (Promega), primed with oligo-dT and with Superscript II (Invitrogen). qPCR was performed by using TaqMan master mix (Applied Biosystems, CA, USA) and first-strand complementary DNA as template. mRNA expression was reported using the 2^−ΔΔCT^ method in two independent experiments. Actin was used as the housekeeping gene.

### Transmission electron microscopy (TEM)

TEM of HK-2 cells treated with AST and/or doxSA was performed as previously described (Gui et al., 2020). Briefly, HK2 cells were fixed in 2.5% glutaraldehyde overnight at 4°C and then in 1% osmium tetroxide for 2 h after collection. The fixed cells were dehydrated in graded series of ethanol, and then infiltrated and embedded in Araldite. Sections (ultrathin 50 nm) were stained with 0.5% lead citrate and 4% uranyl acetate before TEM for the mitochondrial ultrastructural observation.

### Western blot analysis

Western blot assay was performed according to the described protocol procedures. HK2 cells on tissue culture dishes were washed twice with cold PBS at 6, 12, 18, and 24 h after AST and/or doxSA treatment. The cells were then lysed with ice-cold RIPA lysis buffer (150 mM NaCl, 50 mM Tris pH 7.4, 0.5% deoxycholic acid, 0.1% SDS, 1% Triton X-100, 2.5 mM EDTA), including protease inhibitors (1 mM, Beyotime, China) for 30 min at 4°C. The supernatant was collected from the lysate after centrifugation and the protein concentration was measured. The proteins were separated by SDS-PAGE and transferred to PVDF membranes. The membranes were blocked for 1 h at room temperature and incubated with different primary antibodies, namely Nrf2 (ab137550, Abcam), p-p62 (ab211324, Abcam), p62 (ab207305, abcam), HO-1 (ab68477, abcam), PDI (PA5-85950, ThermoFisher Scientific), KU-70 (PA5-27538, ThermoFisher Scientific) for overnight at 4°C. The membranes were washed and then incubated with secondary antibodies conjugated to horseradish peroxidase (HRP, Beyotime, China) for 1 h at room temperature. Chemiluminescence was used to detect the proteins and normalize the bands to PDI levels (cytoplasmic) and KU-70 levels (nuclei). ImageJ analysis software was used to measure the density of the bands (National Institutes of Health).

### Statistical analysis

Statistical analysis was performed in GraphPad Prism 5 (GraphPad) software. The data has been presented as mean with ±SD. Two-sample *t*-test was used to analyze the percentage of apoptosis between the different groups. For other data in the article, one-way ANOVA was used and *p* < 0.05 indicated the significance level.

## Results

### Abnormal 1-deoxysphingolipid levels exert cytotoxic effects leading to the mitochondrial damage and oxidative stress in renal tubule cells

**FIGURE 1:**
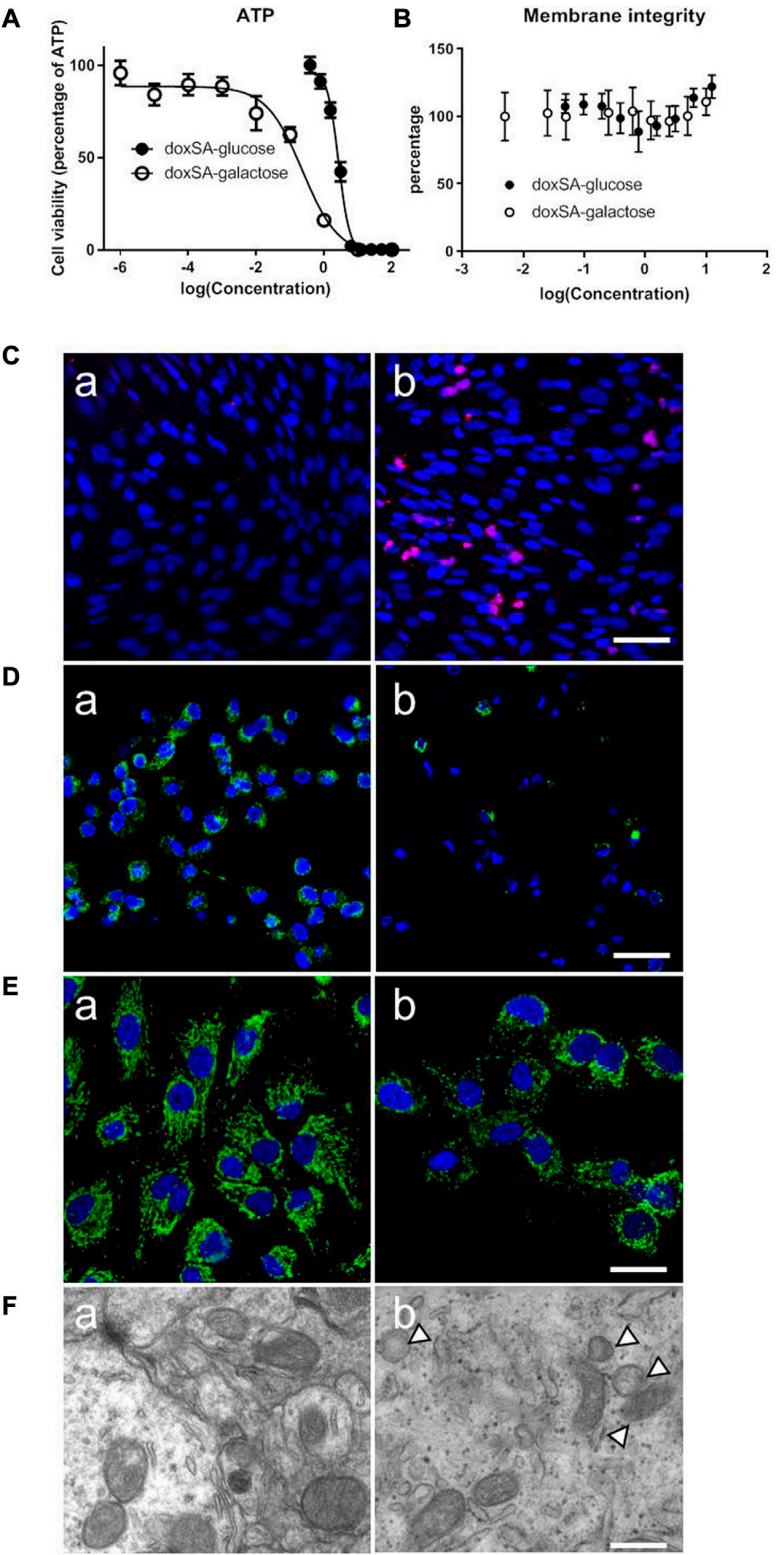
Abnormal 1-deoxysphingolipid levels exert cytotoxic effects in HK-2 cells. **(A)** doxSA induced cell ATP depletion dose-dependently in the culture medium containing glucose or the same amount of galactose, in which the ATP production was switched to be mitochondrial dependent. HK-2 cells in the glucose-free medium were more sensitive to doxSA than those in the medium with the normal glucose. **(B)** doxSA-induced ATP depletion was cell membrane integrity independent. Representative images of **(C)** TUNEL staining, **(D)** rhodamine uptake, and **(E)** immunostaining for the mitochondrial membrane protein Tom20, of HK-2 cells after **(a)** vehicle **(b)** or doxSA (5 μM) treatment for 24 h. Scale bar = 50 μm. **(F)** Representative TEM images of HK-2 cells after **(a)** vehicle **(b)** or doxSA (5 μM) treatment for 24 h. Scale bar = 150 nm. Arrowheads indicate abnormal mitochondria.

Our previous study has indicated that the levels of 1-deoxysphingolipids were increased in both patients and animals suffering from CKD. However, the potential effects of 1-deoxysphingolipids on kidney tubular cells have still not been examined yet. Since 1-deoxysphingolipids are toxic to several types of cells, including hepatocytes and neurons (Alecu et al., 2017; Gai et al., 2020), we first examined the mitochondrial toxic effects of 1-deoxysphinganine on the kidney tubule cells. As shown in Figure 1A, doxSA induced cell ATP depletion in a dose-dependent fashion. HK-2 cells in the glucose-free medium were found to be more sensitive to doxSA as compared to those in the medium with the normal glucose. Moreover, doxSA-induced ATP depletion was cell membrane integrity independent with Figure 1B, indicating that such ATP depletion was more likely to occur because of mitochondria-mediated cell apoptosis than necrosis.

Next, HK-2 cells were subjected to doxSA treatment were analyzed for apoptosis and mitochondrial damage. As shown in Figure 1C, the treatment with doxSA (5 μM) significantly increased TUNEL-positive cells after 24 h. The level of rhodamine uptake and immunostaining for the mitochondrial membrane protein Tom20 indirectly indicated the mitochondrial transmembrane potential and mitochondrial number, which were used to examine the doxSA-induced mitochondrial toxicity in HK2 cells. It was observed that the treatment with doxSA significantly reduced rhodamine uptake and Tom20 expression compared with control after 24 h (Figures 1D, E). doxSA exposure also resulted in abnormal mitochondrial ultrastructure manifested as swelling and collapse of cristae as compared with the vehicle controls (Figure 1F).

**FIGURE 2:**
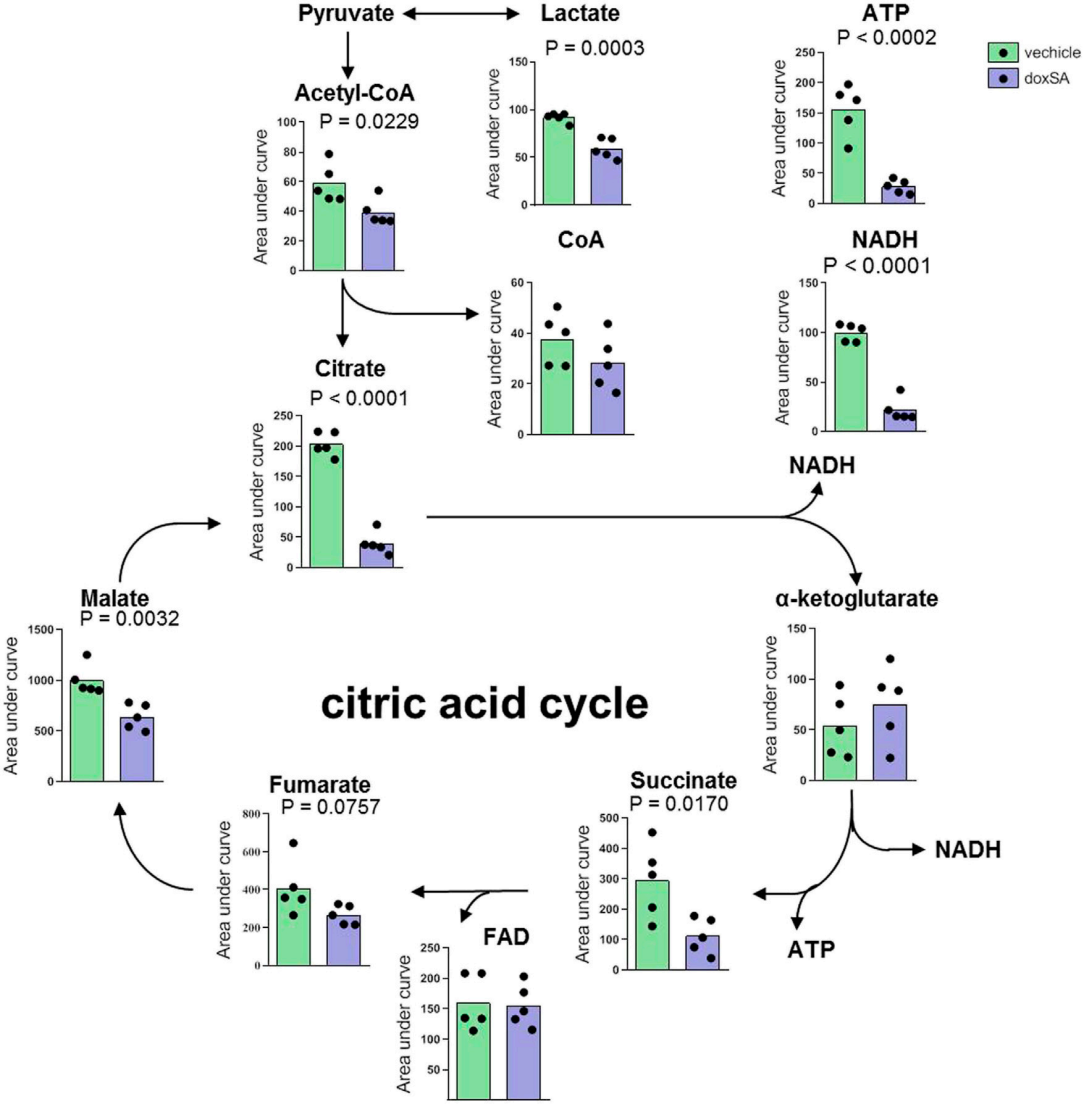
Intracellular citrate cycle-related metabolite profile after doxSA exposure. HK-2 cells were exposed to the vehicle or doxSA at the extracellular concentration of 1 μM for 24 h. The quantitative analysis of the differentially expressed metabolites related to citrate cycle and ATP synthesis.

Thereafter, upon inspecting the metabolomes of mitochondria-related ATP depletion, the intracellular levels of citrate cycle-related metabolites were examined by untargeted metabolomics after the exposure to doxSA (Figure 2). The analysis of identified metabolites revealed that exposure to doxSA significantly reduced lactate, acetyl-CoA, citrate, succinate, malate, and NADH (Figure 2). In addition, quantification of ATP, the final product of the mitochondrial citrate cycle, was substantially reduced in HK-2 cells subjected to doxSA treatment.

### AST can protect HK-2 cells from doxSA-induced mitochondrial damage

**FIGURE 3:**
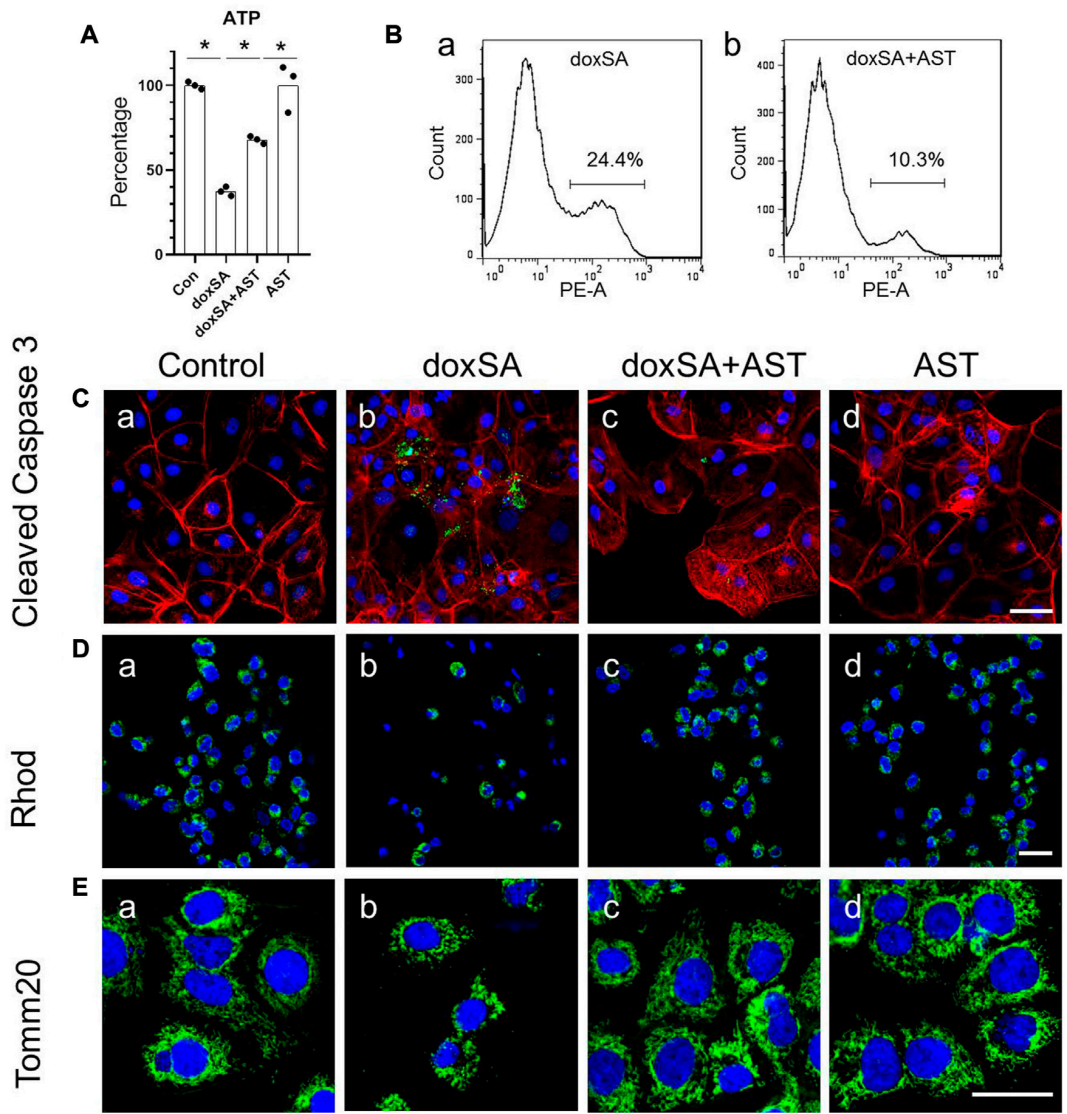
AST protected HK-2 cells from doxSA-induced mitochondrial damage. **(A)** doxSA-induced ATP depletion, **(B)** percentage of annexin-positive apoptosis in cells with (a) doxSA treatment and (b) doxSA treatment with AST. Representative images of **(C)** immunostaining for cleaved Caspase 3, **(D)** rhodamine uptake and **(E)** immunostaining for Tom20 in **(a)** control, **(b)** doxSA (5 μM)-treated, **(c)** doxSA (5 μM) +AST (200 μM)-treated, and **(d)** AST (200 μM)-treated cells have been shown. Scale bar = 100 μm.

AST is a well-known natural compound that has been used in the treatment of kidney disease and has anti-inflammatory and anti-oxidative stress effects (Zhang et al., 2020). To examine its potential protective effect against doxSA-induced oxidative stress and mitochondrial damage in CKD, we first co-treated HK-2 cells with AST and doxSA. doxSA induced ATP depletion (Figure 3A), significantly higher number of annexin-positive apoptosis cells (Figure 3Ba), increase of cleaved caspase 3 (Figure 3Cb), reduced rhodamine uptake (Figure 3Db) as well as less number of Tom20-positive mitochondria (Figure 3Eb). Co-treatment with AST ameliorated doxSA-induced ATP depletion and apoptosis (Figures 3A, Bb), inhibited cleaved caspase 3 (Figure 3Cc), and restored rhodamine uptake as well as tom20-positive mitochondria (Figures 3Dc, Ec). The single maximum dose of AST (200 mg/ml) did not cause substantial apoptosis and death (*p* > 0.05 compared with the Ctrl group) (Figures 3A, C).

### AST reduced oxidative stress and enhanced antioxidant capacity in CKD rats

**FIGURE 4:**
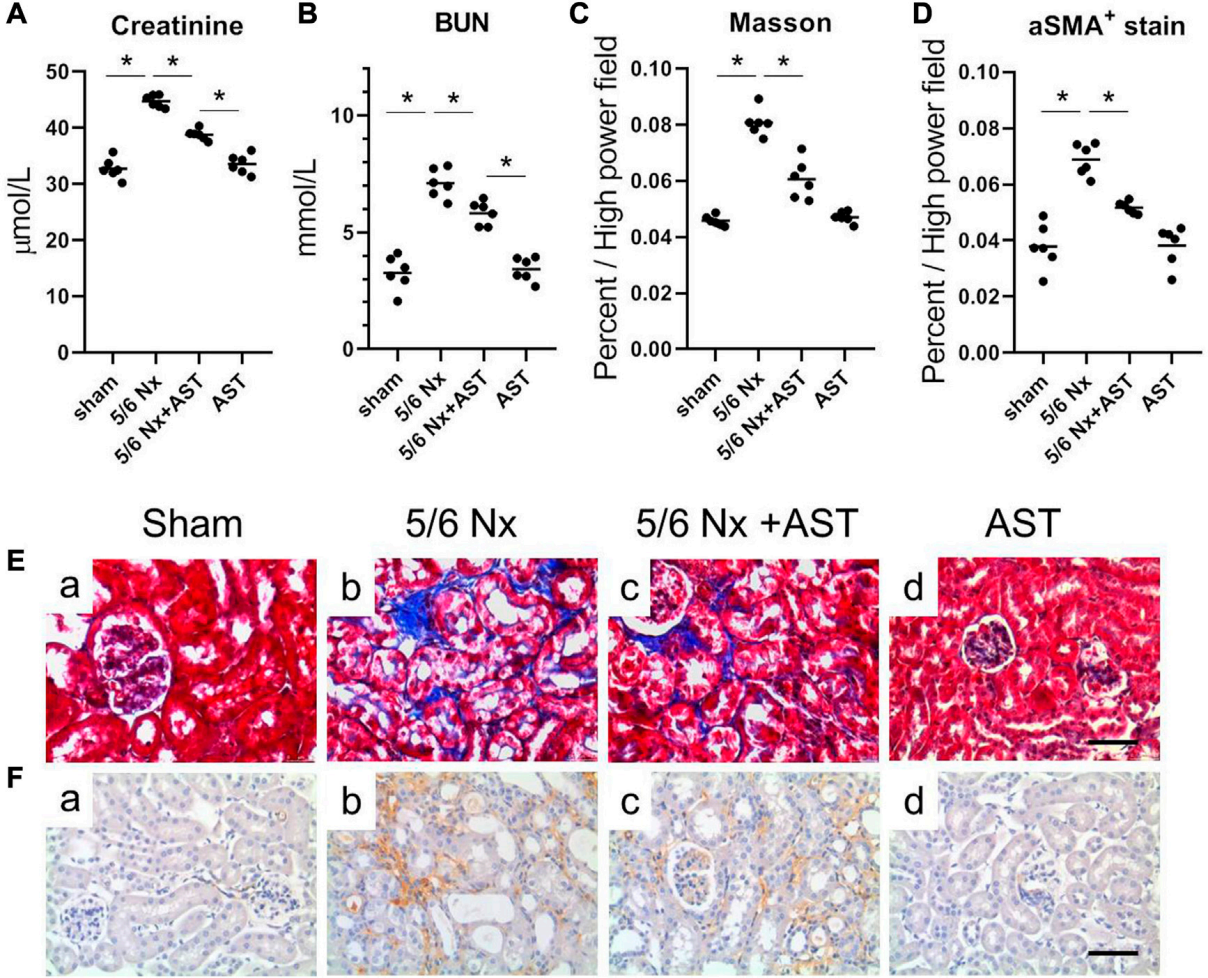
AST protected kidney against 5/6-induced injury. The serum samples were collected for the measurements of **(A)** creatinine, **(B)** BUN. **(C,D)** Quantitative analysis of the **(C)** fibrosis positive area and **(D)** aSMA positive area per high power field. **(E,F)** Representative images showing Masson staining and immunostaining for aSMA in the renal sections from **(a)** sham, **(b)** 5/6 Nx, **(c)** 5/6 Nx + AST and **(d)** AST groups (scale bar 100 μm).

Increased urinary albumin and FFA exposure may increase 1-deoxysphingolipid levels in proximal tubules in CKD patients and 5/6 Nx-treated animal models of CKD and can lead to tubular damage, oxidative stress, and mitochondrial dysfunction (Gui et al., 2021). We next examined the potential protective effects of AST against oxidative stress and mitochondrial damage in CKD using AST in rats with 5/6 Nx-induced CKD. *In vivo*, the renal function assessment was performed by serum creatinine and BUN. The levels of serum creatinine and BUN were significantly increased in 5/6 Nx rats and were both attenuated in rats treated with AST (Figures 4A, B). Serum BUN and creatinine levels in the AST sham group (sham-AST) were found to be the same as in the sham group.

Renal remodeling was assessed by Masson staining of interstitial fibrosis and immunostaining for α-SMA expression. The results of both Masson staining and immunostaining for α-SMA expression revealed more extensive interstitial fibrosis in the kidney of 5/6 Nx rats compared to the sham group (Figures 4Eb, Fb). AST markedly reduced the severity of interstitial fibrosis (Figures 4Ec, Fc). The percentage of Masson-positive and α-SMA-positive areas was greater in 5/6 Nx rats as compared to those in those of the sham group (Figures 4C, D). Both were significantly reduced in 5/6 Nx rats supplemented with AST (Figures 4C, D).

**FIGURE 5:**
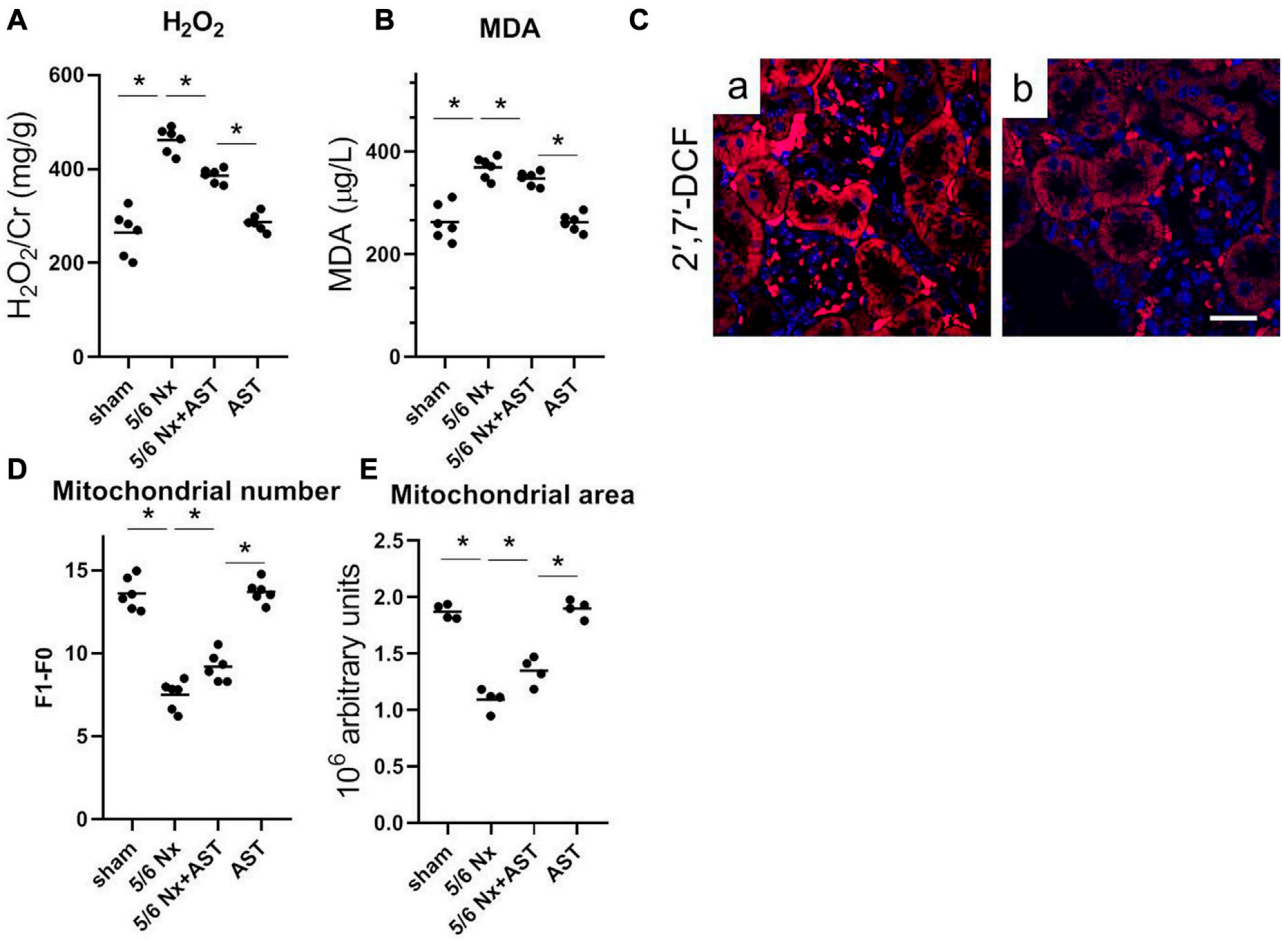
AST reduced 1-deoxySL-induced oxidative stress in 5/6 Nx rats. Quantitative analysis of **(A)** urinary H2O2 and **(B)** serum MDA from rats in sham, 5/6 Nx, 5/6 Nx + AST and AST groups. n = 6/group. The data shown are means ± SD, one-way ANOVA with Bonferroni’s test. **(C)** Representative images showing oxidative stress with 2′,7′-DCF staining on the fresh renal sections from **(a)** 5/6 Nx and **(b)** 5/6 Nx + AST groups (scale bar 50 μm). Quantitative analysis of electron microscopy images of **(D)** mitochondrial number and **(E)** mitochondrial area. n = 6 mice/group. The data shown are means ± SD, one-way ANOVA with Bonferroni’s test. *p < 0.05.

Oxidative stress has been related to CKD and 1-deoxysphingolipid increase. To examine whether AST can effectively reduce 1-deoxySL through affecting oxidative stress in the kidney, the levels of the various oxidative stress markers were examined. It was observed that compared with the sham group, 5/6 Nx significantly increased both H_2_O_2_ and MDA (Figures 5A, B). However, the levels of both indicators were lower with AST co-treatment, thus suggesting its antioxidant effects. Next, a specific fluorescent indicator for ROS, H2DCFDA, was used to detect the ROS production. ROS-induced oxidation can promote the conversion of non-fluorescent H2DCFDA to highly fluorescent 2′,7′-dichlorofluorescein (2′,7′-DCF). Fluorescence in 5/6 Nx kidney tissue was found to be positive in the tubular cells (Figure 5Ca), indicating higher ROS production. AST co-treatment resulted in a significant decrease in 2′,7′-DCF fluorescence in 5/6 Nx rats, indicating lower levels of oxidative stress (Figure 5Cb). This result was consistent with the reduction of the mitochondrial number and area in 5/6 Nx rats by electron microscopy (Figures 5D, E). In contrast, the mitochondrial numbers and their areas after 5/6 Nx were closer to normal with AST treatment (Figures 5D, E).

### AST inhibited oxidative stress through p62 phosphorylation and Nrf2 activation

The p62/NRF2 pathway has been reported to play an important role in antioxidant regulation in various studies (Fujiki et al., 2019; Hu et al., 2019; Zhou et al., 2021). To address whether the p62/NRF2 pathway can mediate AST–induced anti-oxidative protection, we evaluated the nuclei translocation of Nrf2 in AST-treated HK-2 cells. 6 h after AST treatment, it was found that expression of Nrf2 protein started to increase in the nuclei of HK-2 cells. Its increase reached peak level around 12 h and returned to normal at 24 h. However, Nrf2 protein level was not significantly changed in the cytoplasm lysis of HK-2 cells with the same treatment, thereby indicating that AST treatment could induce Nrf2 activation in HK-2 cells (Supplementary Figure S1).

**FIGURE 6:**
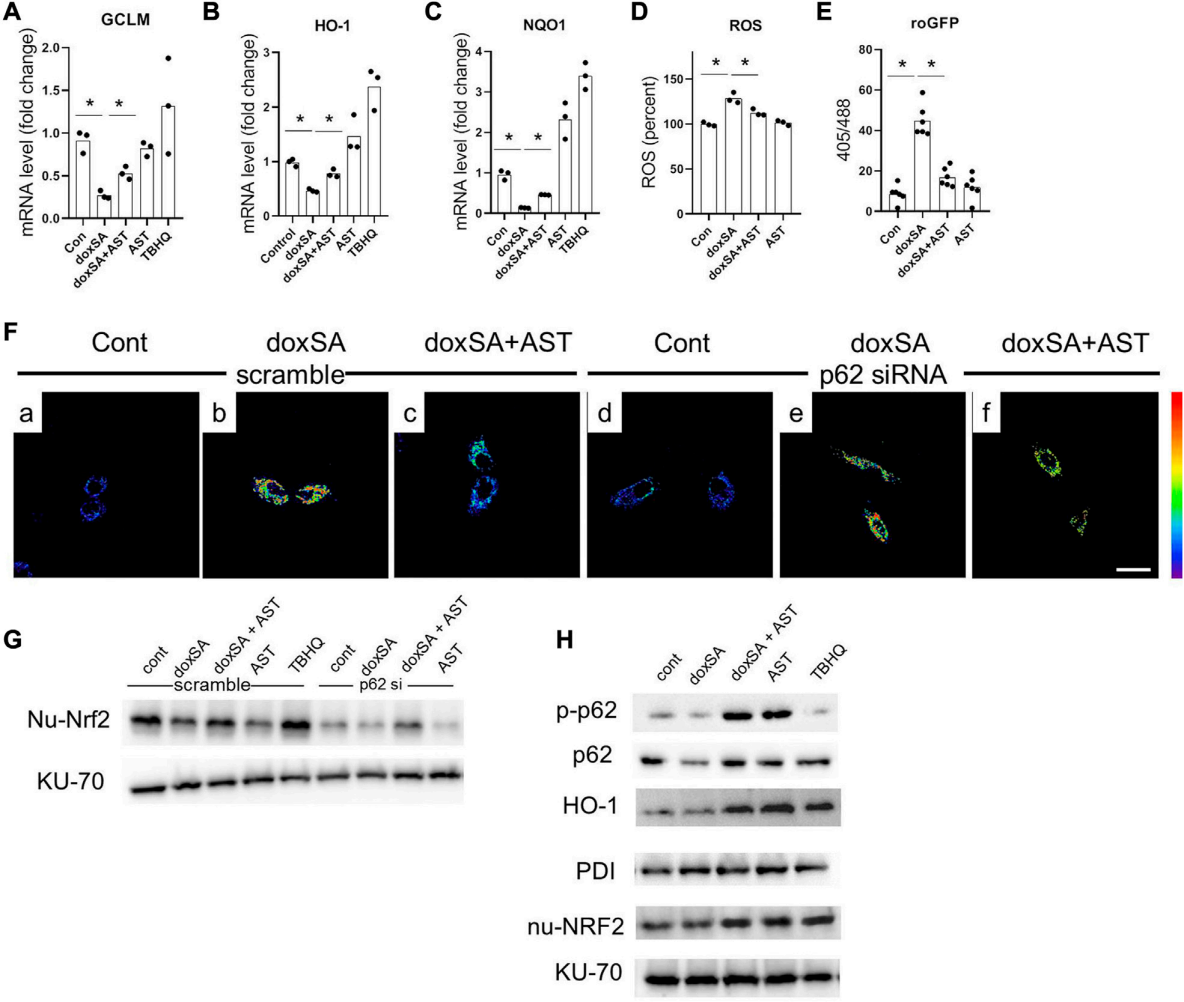
AST inhibited oxidative stress through modulating p62-NRF2 signaling pathway. mRNA expression of **(A)** GCLM, **(B)** HO-1, and **(C)** NQO-1 in the HK-2 cells after exposure to the different treatments. n = 6/group. The data shown are means ± SD, one-way ANOVA with Bonferroni’s test. Quantitative analysis of **(D)** intracellular ROS, and **(E)** roGFP in the HK-2 cells with different treatments. n = 6/group. The data are means ± SD, one-way ANOVA with Bonferroni’s test. **(F)** Representative images of mitochondrial ROS sensing in HK-2 cells in **(a–c)** scramble siRNA and in **(d–f)** p62 siRNA-transfected HK-2 cells after exposure to the different treatments (scale bar 100 μm). **(G)** Representative WB images for the nuclear Nrf2 (nu-Nrf2) and Ku-70 of HK-2 cells after control, doxSA (5 μM)-treated, doxSA (5 μM) +AST (200 μM)-treated, and AST (200 μM)-different treatments. **(H)** Representative WB images for phospho-p62 (p-p62), p62, HO-1, PDI, nuclear Nrf2 (nu-Nrf2) and Ku-70 of HK-2 cells with different treatments.

To further address the role of p62/NRF2 pathway in AST–induced anti-oxidative effect, we evaluated the antioxidant genes downstream of p62/NRF2 pathway in doxSA-induced mitochondrial damage in HK-2 cells. It was observed that 24 h treatment with doxSA reduced the expression of different Nrf2 downstream genes (*GCLM, H O -1*, and *NQ O 1*) (Figures 6A–C), induced ROS production (Figure 6D), particularly that of mitochondrial ROS (Figure 6E). AST co-treatment promoted the levels of genes involved in antioxidant defense (Figures 6A–C), ameliorated the level of oxidative stress, both in cells and in the mitochondria (Figures 6D, E, respectively). AST treatment alone could also induce increases of HO-1 and NQO1, which was consistent with Supplementary Figure S1.

We next investigated whether AST-mediated Nrf2 antioxidant pathway was p62 dependent. For this, HK-2 cells with stable mito-roGFP expression were constructed and then transiently transfected with either scrambled siRNA or p62 siRNA. Fluorescent image showed a higher ratio of oxidized roGFP in mitochondria of HK-2 cells transfected with scrambled siRNA after doxSA treatment (Figure 6Fb). The same cells when co-treated with doxSA and AST displayed a less ratio of oxidation in mitochondria (Figure 6Fc). However, HK-2 cells transfected with p62 siRNA exhibited indiscriminate ratio of sensor oxidation after doxSA treatment without/with AST (Figures 6Fe, f, respectively), thus indicating that the AST-mediated antioxidant effect in mitochondria was p62 dependent. The p62 dependence of Nrf2 induction in response to AST was further compared to that in response to TBHQ, which can activate Nrf2 in a p62 independent pathway (Chen et al., 2022). Immunoblot analysis showed an increase of nuclear-Nrf2 in HK-2 cells transfected with scrambled siRNA group and then treated doxSA without/with AST (Figure 6G). When p62 was knocked down, Nrf2 nuclei translocation by AST was completely abrogated (Figure 6G). In comparison, TBHQ induced rapid Nrf2 translocation in HK-2 cells transfected with p62 siRNA. Furthermore, AST treatment induced phosphorylation of p62 in HK-2 cells, in parallel with Nrf2 translocation and its downstream target HO-1 (Figure 6H). Taken together, it could be concluded that AST can ameliorate doxSA-induced oxidative stress through modulation of p62 phosphorylation and Nrf2 nuclear translocation.

## Discussion

We have previously reported that intracellular oxidative stress was related to the increase of 1-deoxysphingolipids in CKD under both *in vivo* and *in vitro* settings (Gui et al., 2021). Abnormal 1-deoxysphingolipid levels have been reported to exert cytotoxic and mitochondrial toxic effects, but the underlying mechanisms of 1-deoxysphingolipids and related oxidative stress processes remain obscure. In this study, we have performed an untargeted metabolomics analysis and a series of functional tests to establish that doxSA could induce mitochondrial ROS following mitochondrial damage and inhibition of mitochondrial energy production. Such mitochondrial impairments were ameliorated upon AST treatment through modulation the p62-NRF2 antioxidant pathway.

Mitochondrial function has been closely related to the maintenance of renal lipolysis and lipogenesis balance, and mitochondrial dysfunction can impair the process of fatty acid oxidation in patients with obesity and metabolic syndrome (Jimenez-Aranda et al., 2014). doxSA-induced mitochondrial damage was observed in HK-2 cells (Figure 1F), which was identical to mitochondrial morphological changes in an animal model of 5/6 Nx rats. Mitochondrial dysfunction has been reported previously to reduce fatty acid oxidation in an animal model of 5/6 Nx rats (Zhao et al., 2012; Sun et al., 2017; Son et al., 2021). AST treatment protected the kidney from mitochondrial damage, and significantly reduced the level of ROS in rats with CKD.

Oxidative stress caused by excessive ROS production and the occurrence as well as progression of CKD have been reported in several prior studies (Fontecha-Barriuso et al., 2022; Rahman et al., 2022). Oxidative stress can induce substantial chronic inflammation often observed in the process of renal injury and fibrosis, ultimately leading to the renal dysfunction. AST was able to protect the kidneys against inflammation (Chen et al., 2018b), and we found that AST alleviated mitochondrial damage in CKD mice by activating the Nrf2 signaling pathway in the kidneys. AST has been previously reported to protect the renal tubular cells from oxidative damage and apoptosis in 5/6 Nx rats, but promoted the expression of hypoxia-inducible factor-alpha (HIF-alpha) protein in CKD-anemic rats (Chen et al., 2019). We analyzed the role of AST in enhancing kidney antioxidant capacity and preventing mitochondrial damage, to further confirm the protective effect of AST on renal tissue. In particular, AST was able maintain the redox balance by activating the p62/NRF2 signaling pathway in CKD rats, thereby underlining its preventive role in renal dysfunction. At the same time, we demonstrated that AST showed therapeutic effect in improving the renal dysfunction according to fibrosis based on the various parameters associated with pro-fibrosis-related factors such as aSMA.

Oxidative stress inevitably occurs in various CKD models. Severe oxidative stress, increased oxidase activity, and ROS accumulation in the remnant kidneys have been demonstrated in rats with 5/6 Nx-induced CKD (Fujihara et al., 2007; Liu et al., 2018). Excessive production of ROS can thereafter damage DNA, lipids, and proteins, and attack, denature, and modify the various structural and functional molecules present in the kidneys, impair antioxidant defenses, and induce renal tissue organ dysfunction, including mitochondria dysfunction. Overproduction of ROS and/or impaired antioxidant defense can regulate oxidative stress (Malhotra and Kaufman, 2007; Cachofeiro et al., 2008). In the present study, 1-deoxySL targeted mitochondria to effectively inhibit mitochondrial function and stimulated oxidative stress. On the other hand, impaired mitochondrial function and excessive ROS levels in the kidney could damage fatty acid oxidation (Jiang et al., 2020; Takemura et al., 2020), thereby resulting in increased accumulation of intracellular fatty acids, including 1-deoxySL (Gui et al., 2021). Moreover, the increased 1-deoxySL levels can further damage the renal tubules and worsen the kidney function.

**FIGURE 7:**
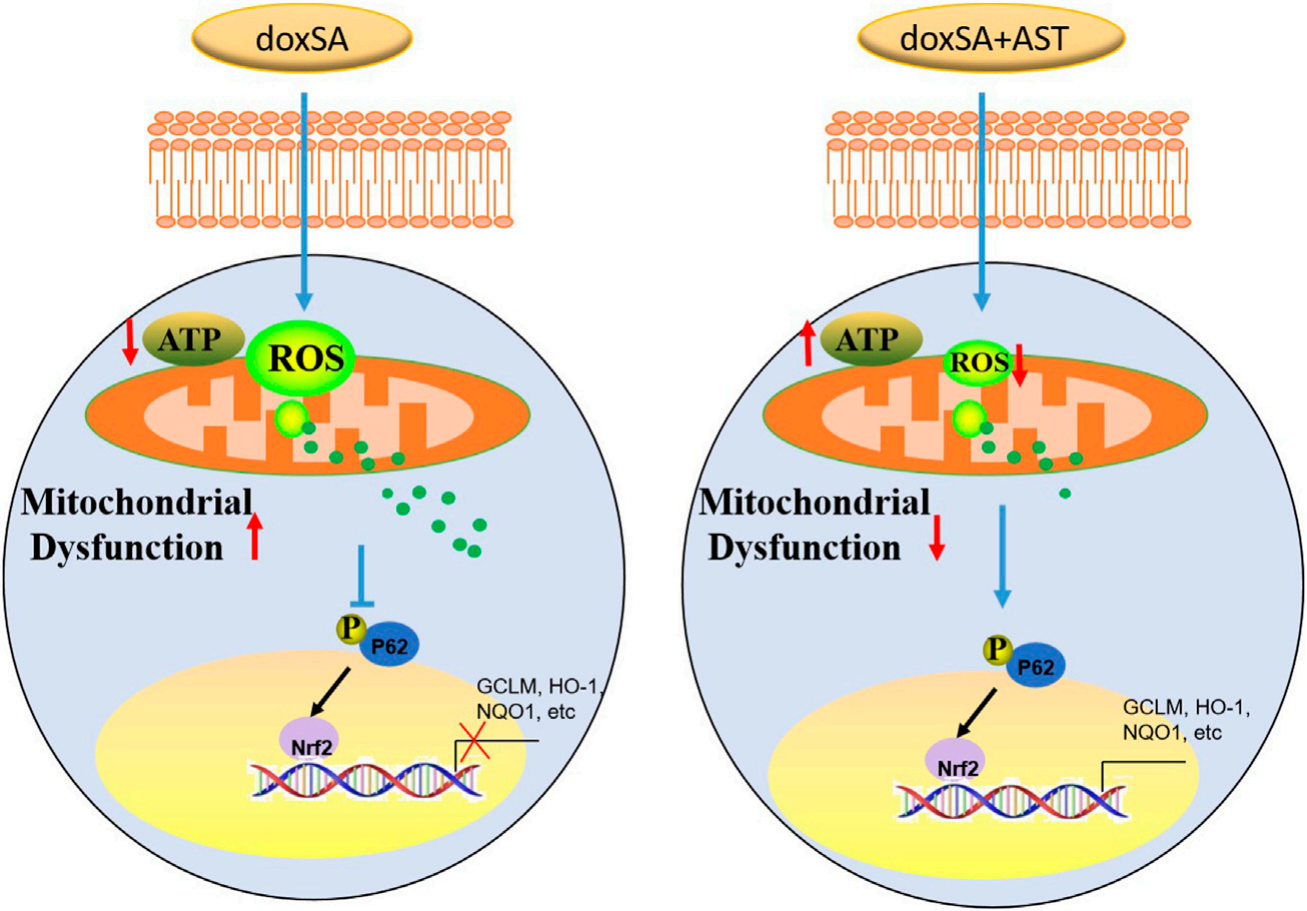
Schemic representation of the signaling involved in this study.

Antioxidant therapy could lead to the development of a novel treatment strategy for CKD (Shah et al., 2007) by protecting the morphological features and functions of the kidneys. The findings of our study indicate that AST can restore the antioxidant function of CKD kidneys in the damaged kidneys. Our study also showed that AST could counteract ROS accumulation as well as oxidative stress, and enhance the activity of Nrf2 signaling pathway. As a master regulator of the various genes responding to antioxidant stress, Nrf2 can encode several detoxifying antioxidants and enzymes (Nezu and Suzuki, 2020). Inhibition of Nrf2 activation can attenuate or even eliminate the expression of genes encoding antioxidants. Nrf2 knockout in rats has been found to exhibit more severe renal structural deformation and dysfunction, leading to different types of CKD such as oxidative stress-induced diabetic nephropathy and lupus-like autoimmune nephritis (Yoh et al., 2001) (Suzuki and Yamamoto, 2015). Thus, it is necessary to elucidate the molecular mechanisms through which AST can regulate the Nrf2 pathway in CKD rats to protect renal function from doxSA-induced oxidative damage. In our study, under exposure to oxidative stress, p62 was silenced, and the transactivation of Nrf2 was attenuated, thereby restraining this protein in the cytoplasm. After AST treatment, Nrf2 can be transactivated, and then translocate into the nucleus. After entering the nucleus, Nrf2 can transactivate the expression of the various cytoprotection-related genes (such as *GCLM*, *HO-1*, and *NQO1*), which can promote cell survival (Figure 7).

## Conclusion

Our present data showed that AST caused a significant upregulation of p62 phosphorylation as well as Nrf2 translocation and then caused the activation of antioxidant defense systems in CKD rats.

## Data Availability

The raw data supporting the conclusion of this article will be made available by the authors, without undue reservation.
