# Supplementary figures and images for: Astragaloside IV alleviates 1-deoxysphinganine-induced mitochondrial dysfunction during the progression of chronic kidney disease through p62-Nrf2 antioxidant pathway
Source: Front Pharmacol. 2023 Mar 24;14:1092475. doi: 10.3389/fphar.2023.1092475 (PMC10079923; doi:10.3389/fphar.2023.1092475)

# Supplementary Figure 1

Cy-Nrf2

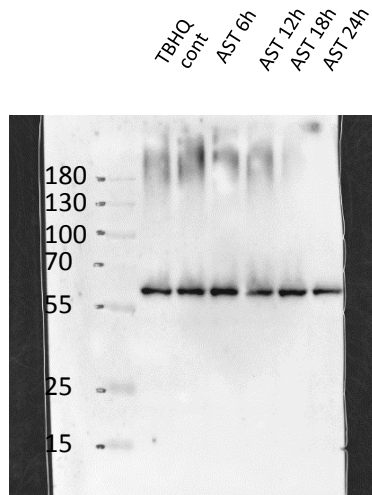

Nu-Nrf2

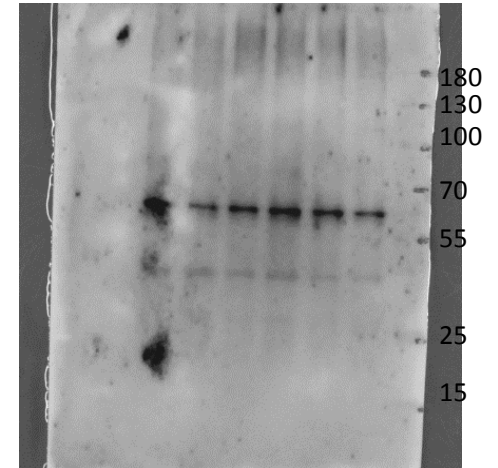

Cy-PDI

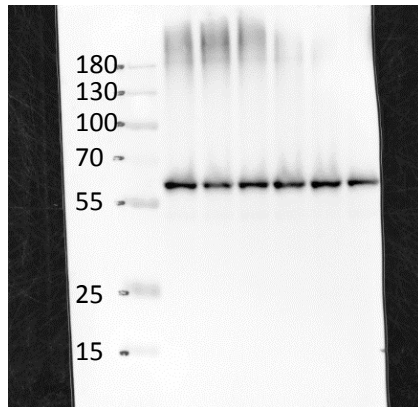

KU-70

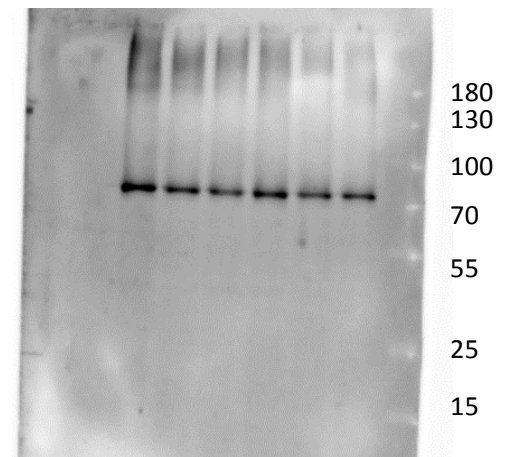

# Figure 6 G

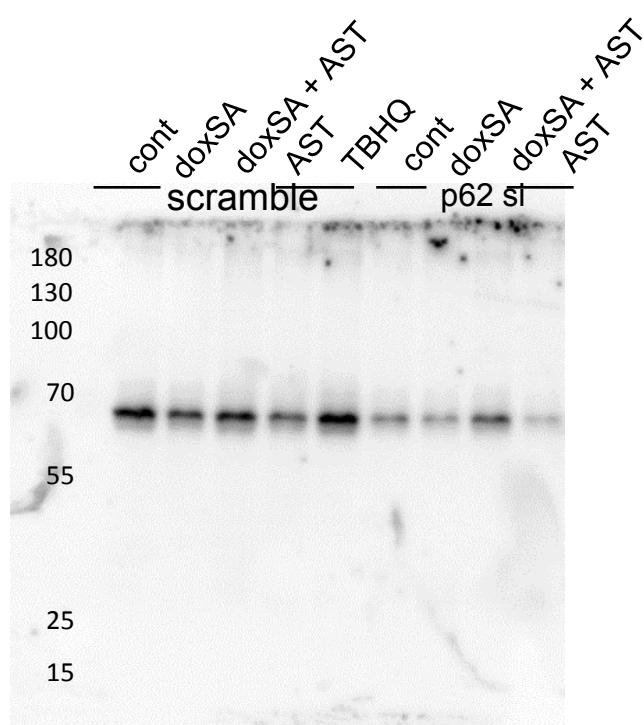

NRF2

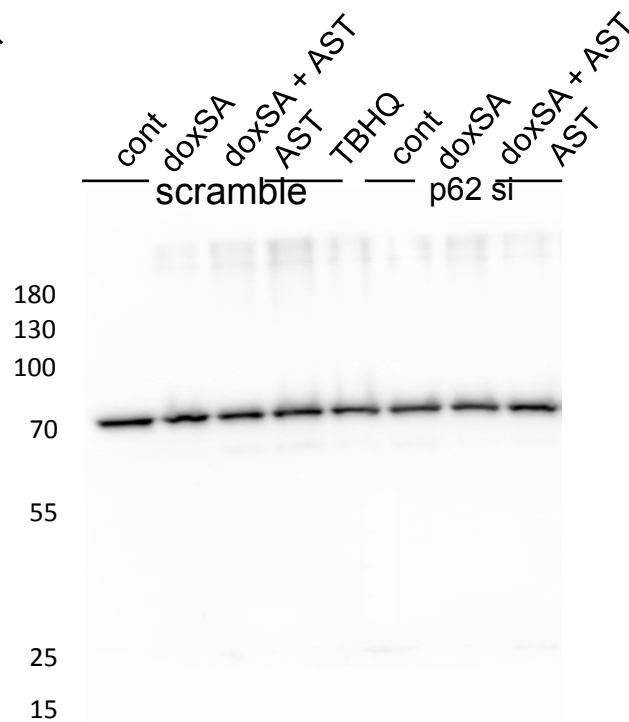

KU-70

# Figure 6 H

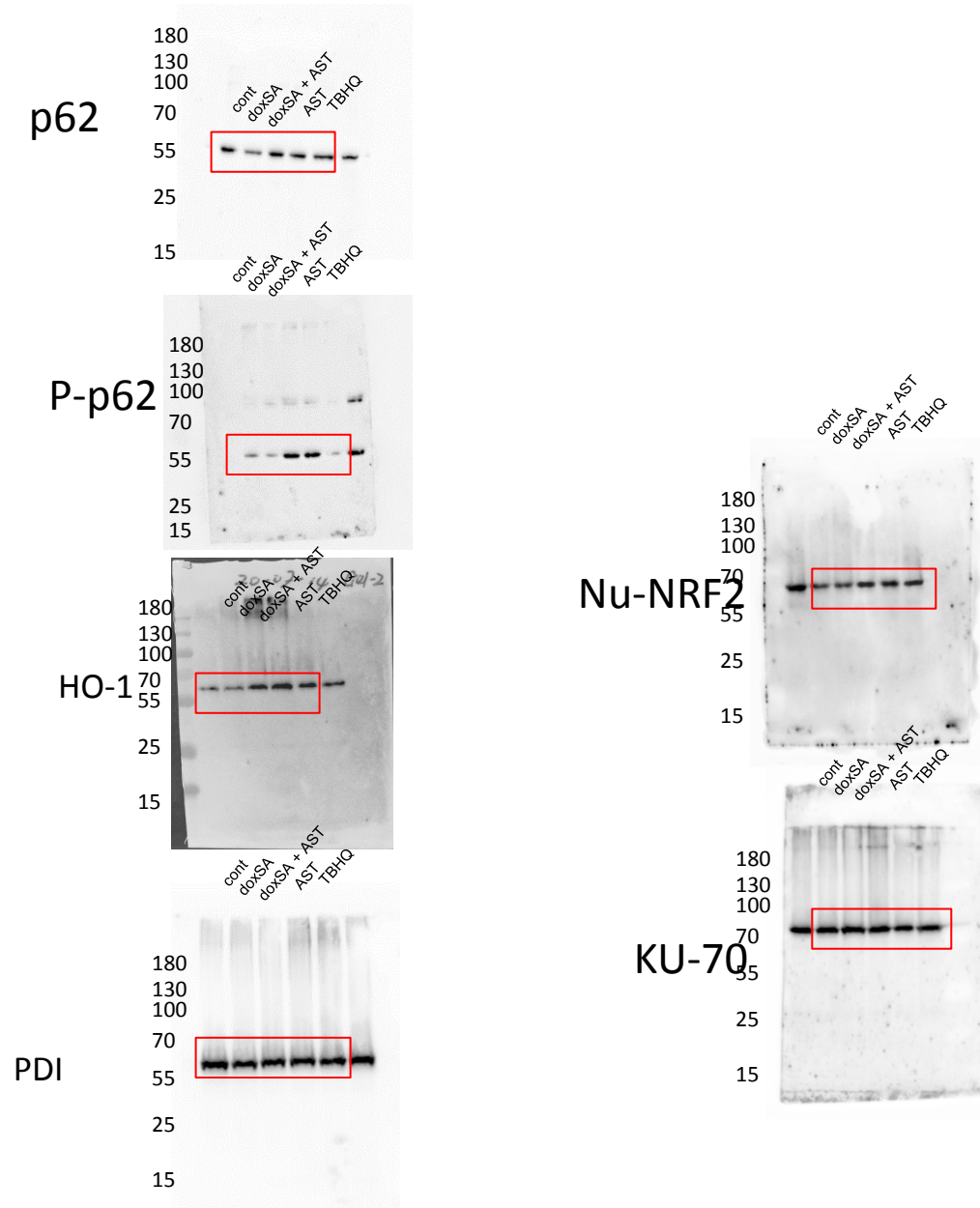

Supplement: Supplementary file 1 [file DataSheet2.PDF]

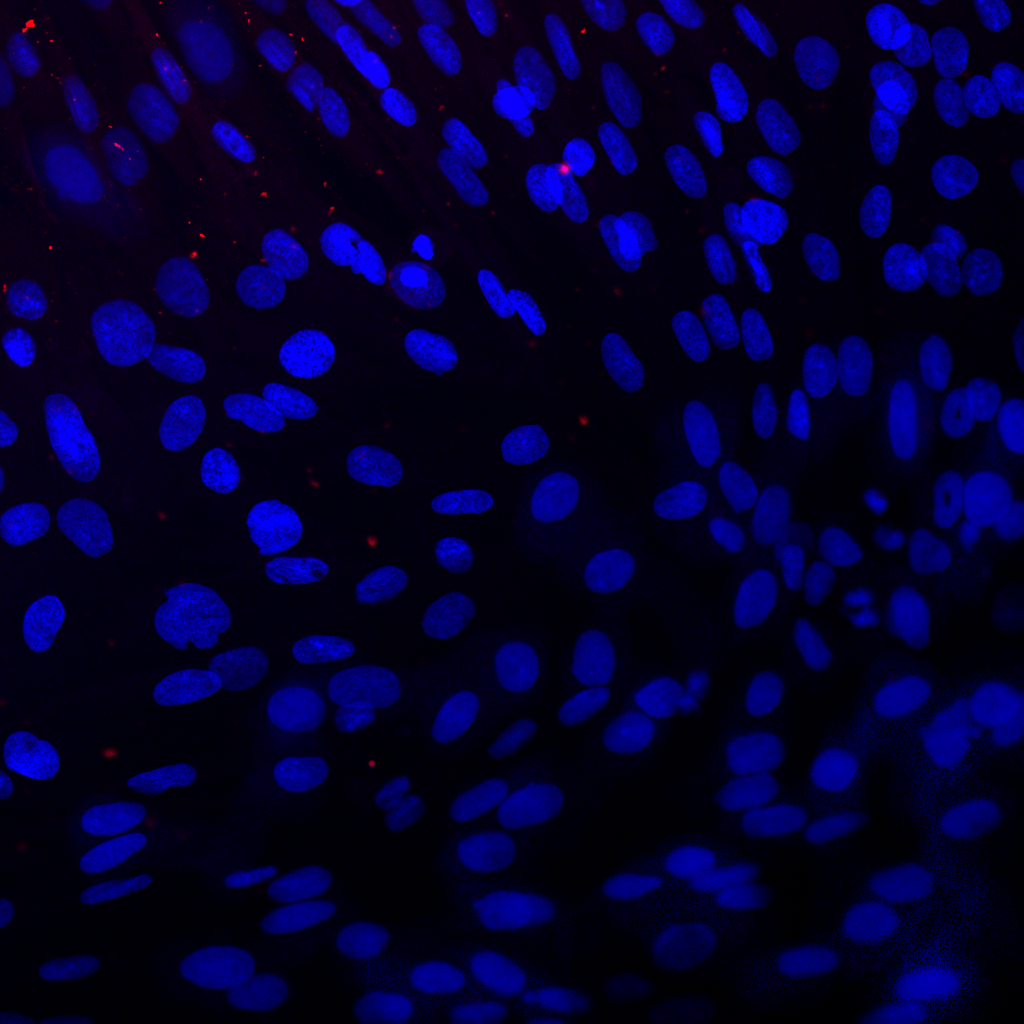

Supplement: Supplementary file 2 [file DataSheet1.ZIP › images/Figure 1/Figure 1 C a.tif]

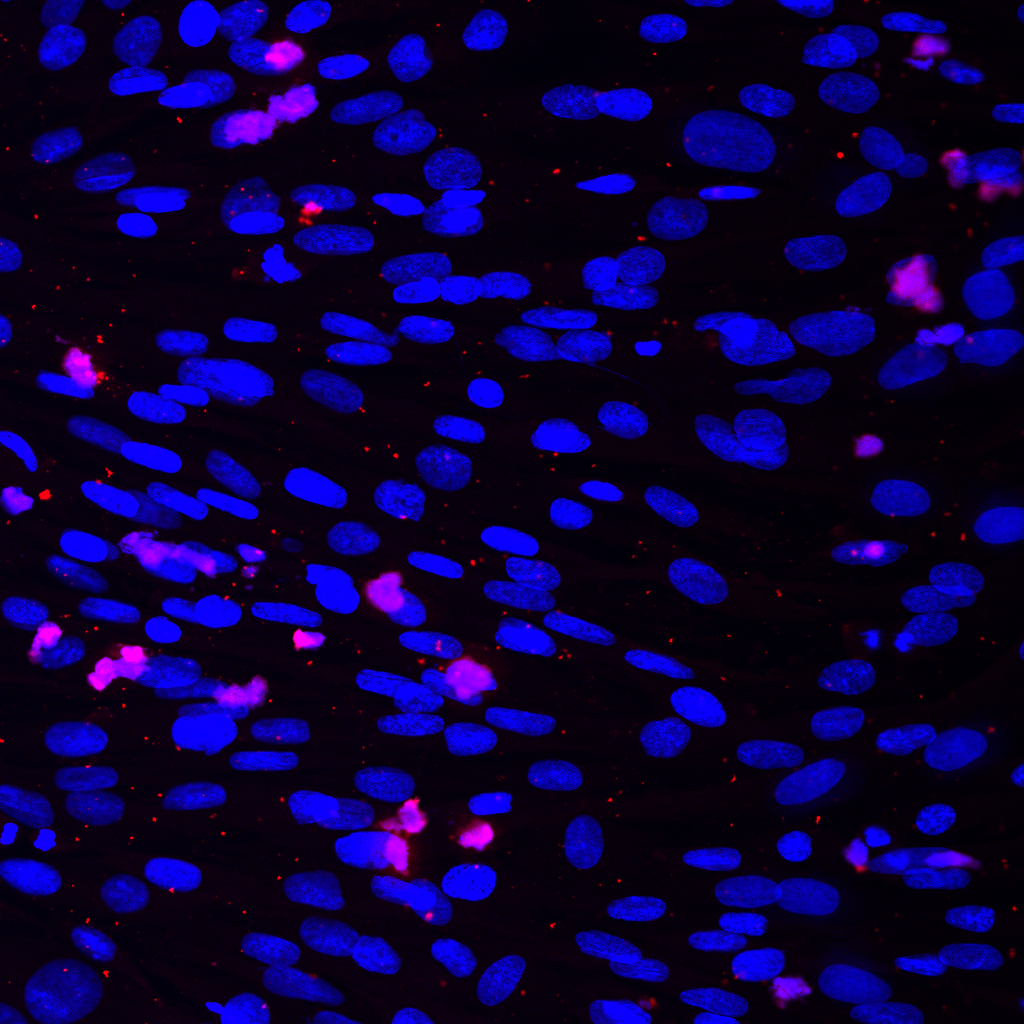

Supplement: Supplementary file 2 [file DataSheet1.ZIP › images/Figure 1/Figure 1 C b.tif]

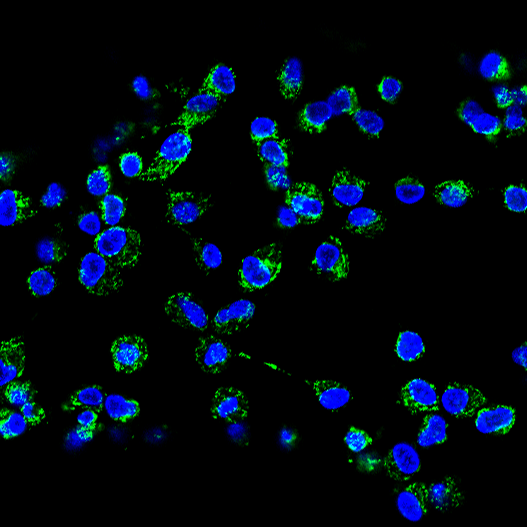

Supplement: Supplementary file 2 [file DataSheet1.ZIP › images/Figure 1/Figure 1 D a.tif]

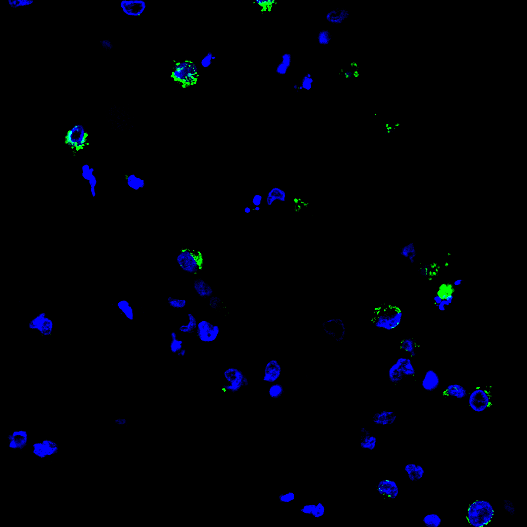

Supplement: Supplementary file 2 [file DataSheet1.ZIP › images/Figure 1/Figure 1 D b.tif]

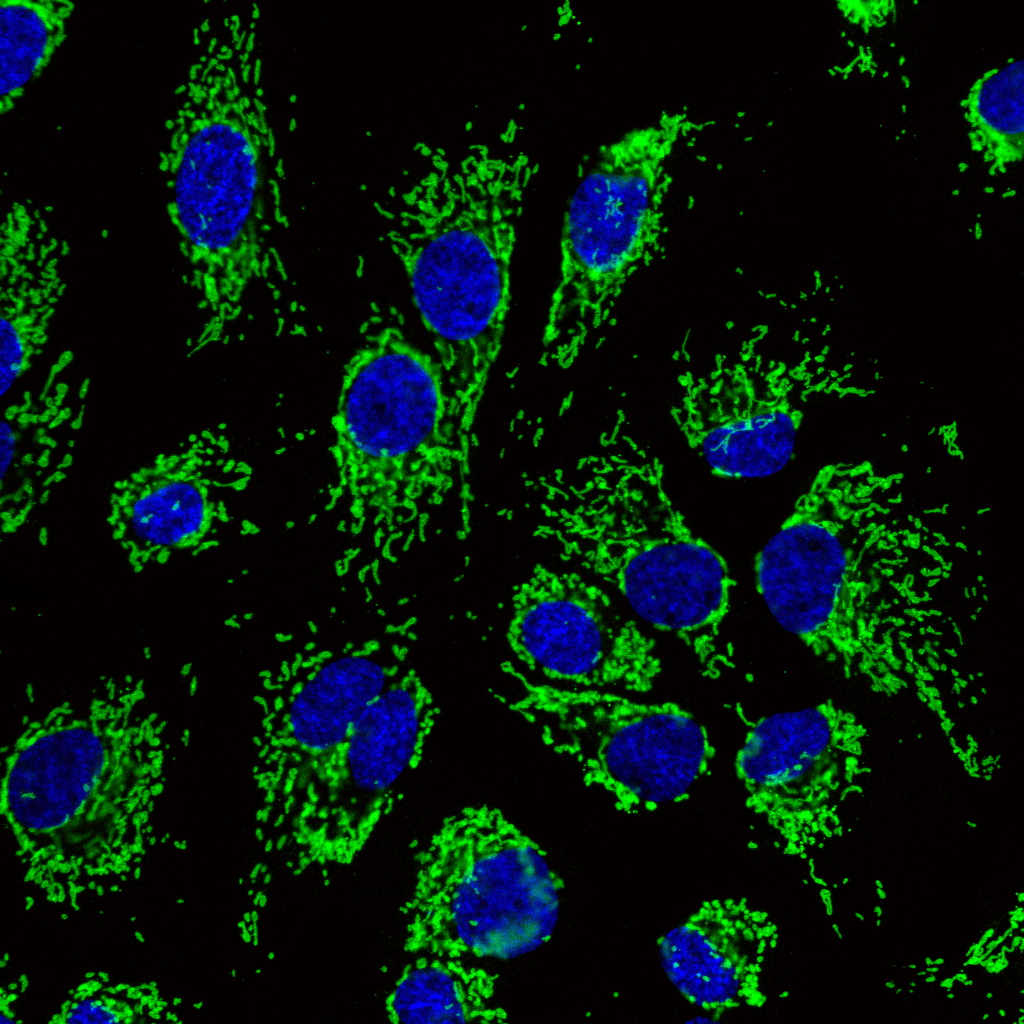

Supplement: Supplementary file 2 [file DataSheet1.ZIP › images/Figure 1/Figure 1 E a.tif]

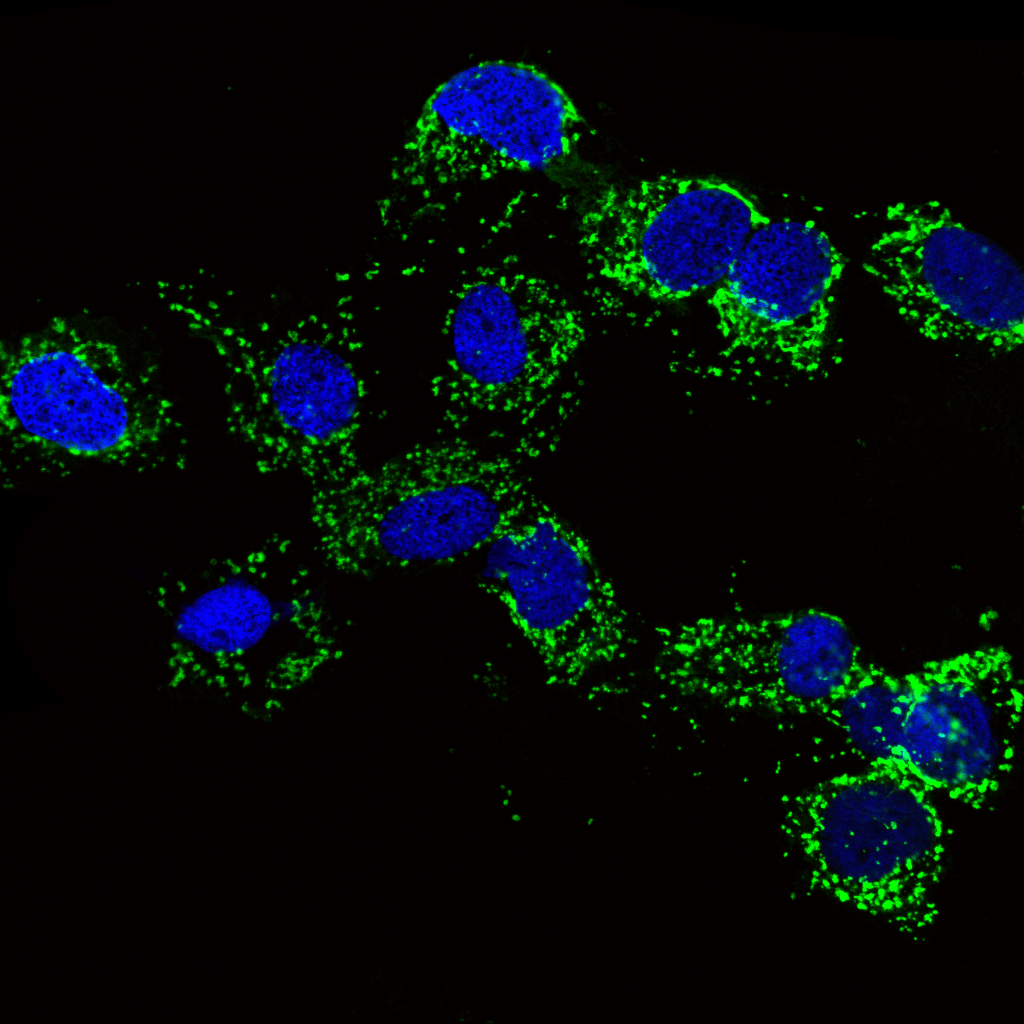

Supplement: Supplementary file 2 [file DataSheet1.ZIP › images/Figure 1/Figure 1 E b.tif]

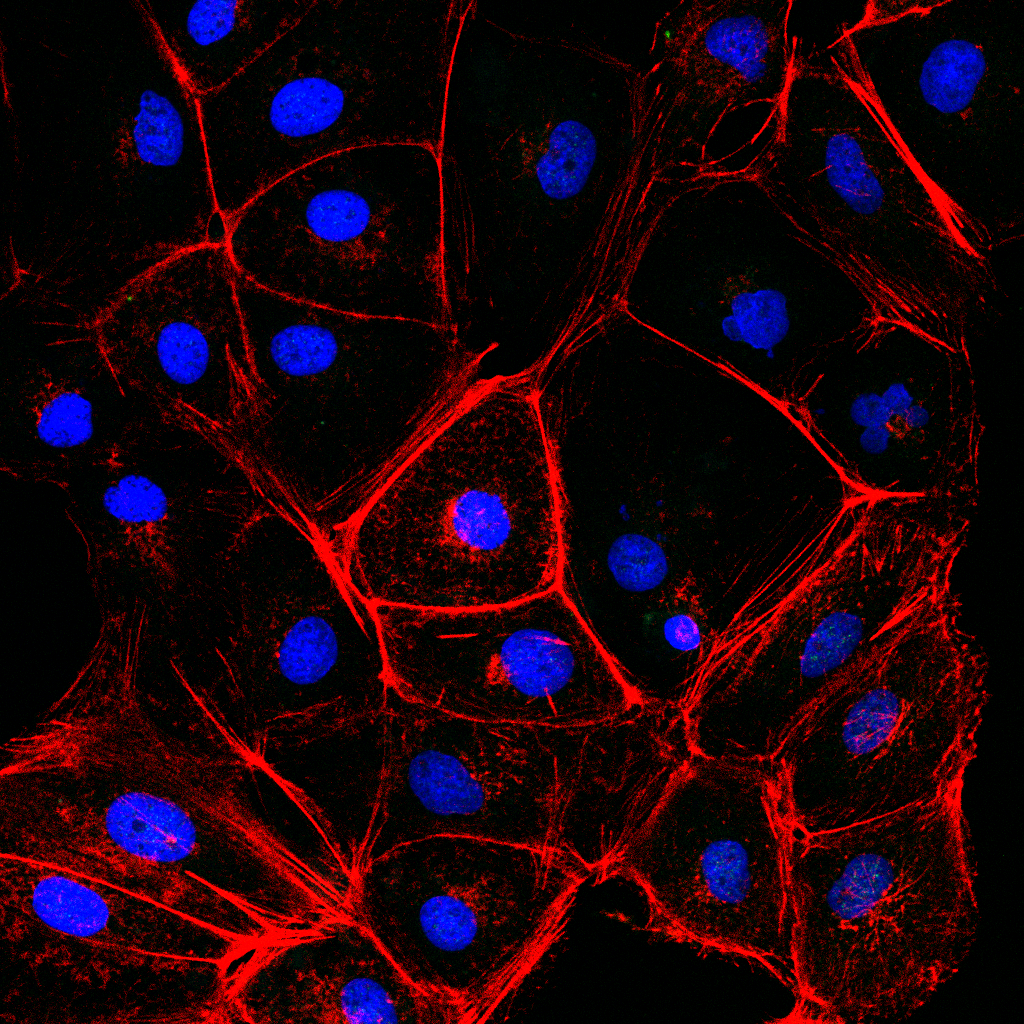

Supplement: Supplementary file 2 [file DataSheet1.ZIP › images/Figure 3/Figure 3 C a.tif]

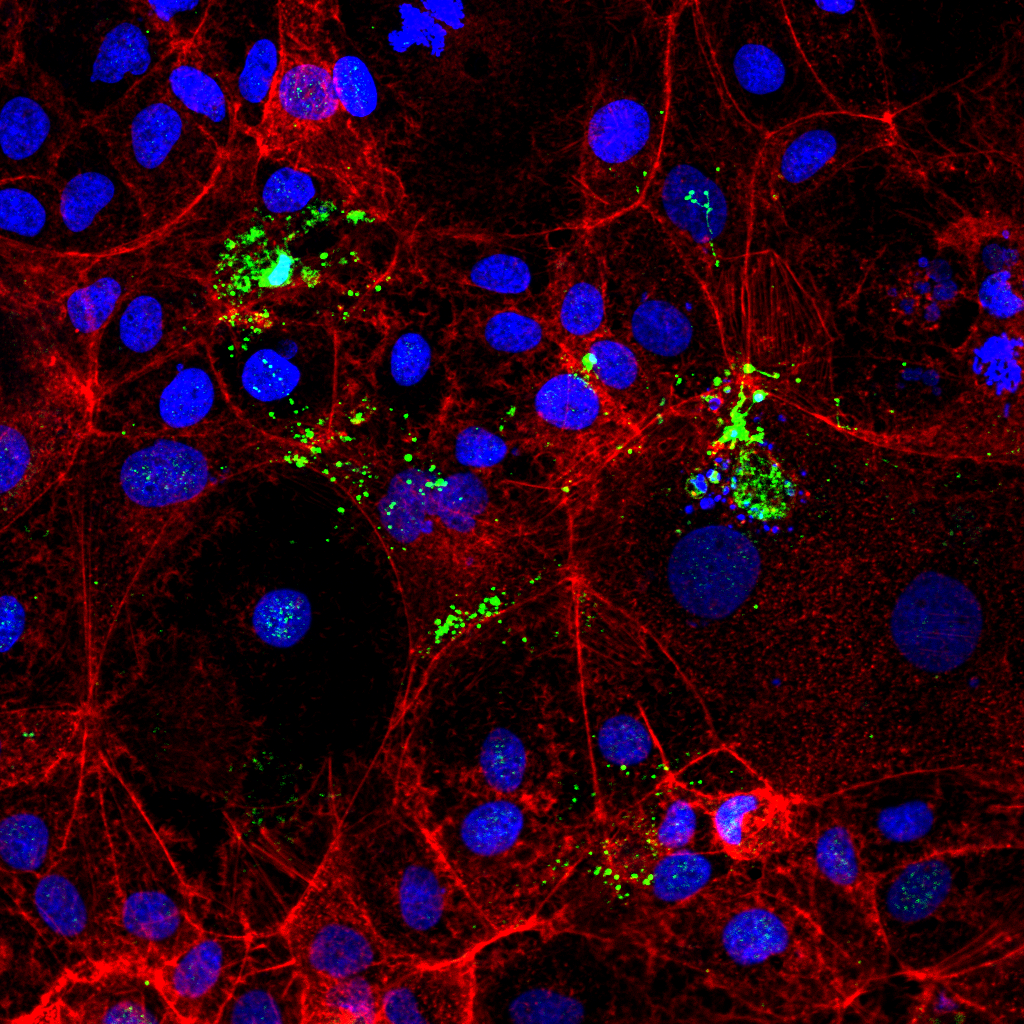

Supplement: Supplementary file 2 [file DataSheet1.ZIP › images/Figure 3/Figure 3 C b.tif]

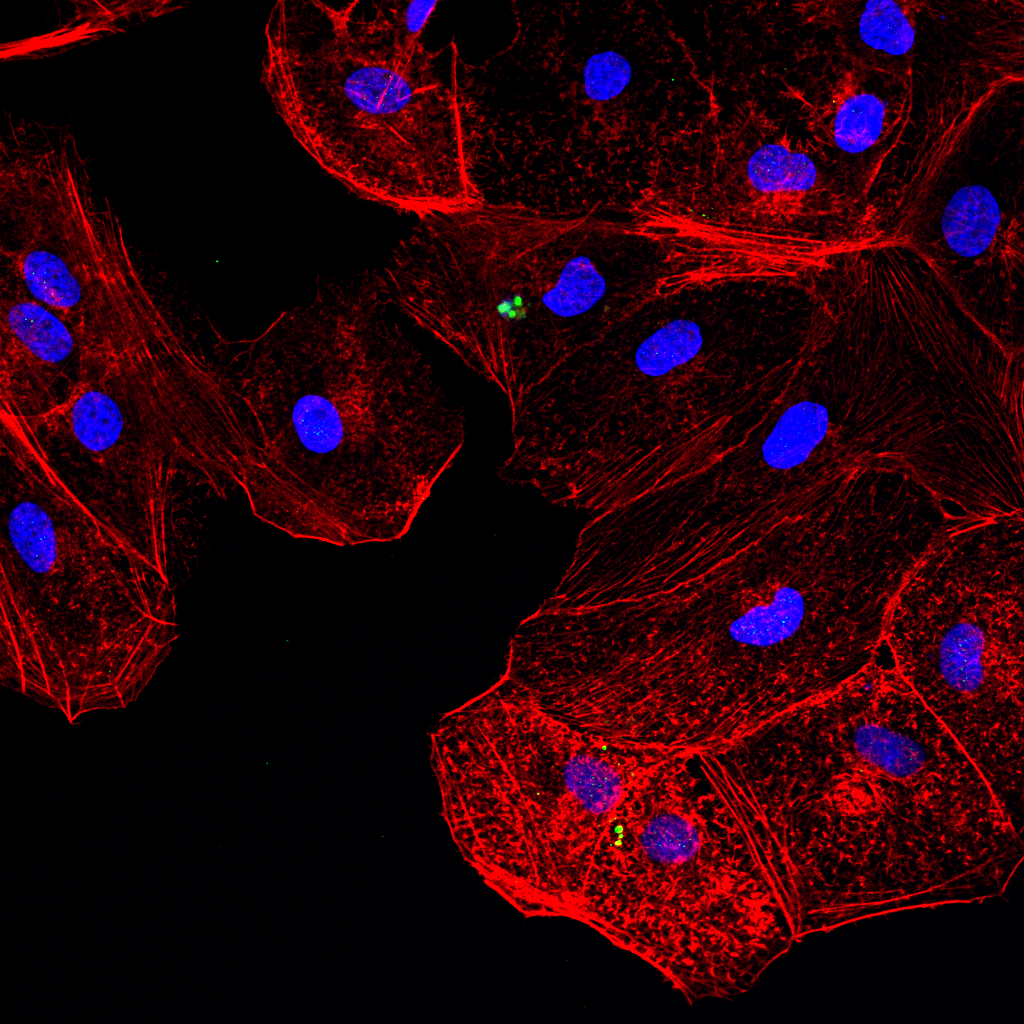

Supplement: Supplementary file 2 [file DataSheet1.ZIP › images/Figure 3/Figure 3 C c.tif]

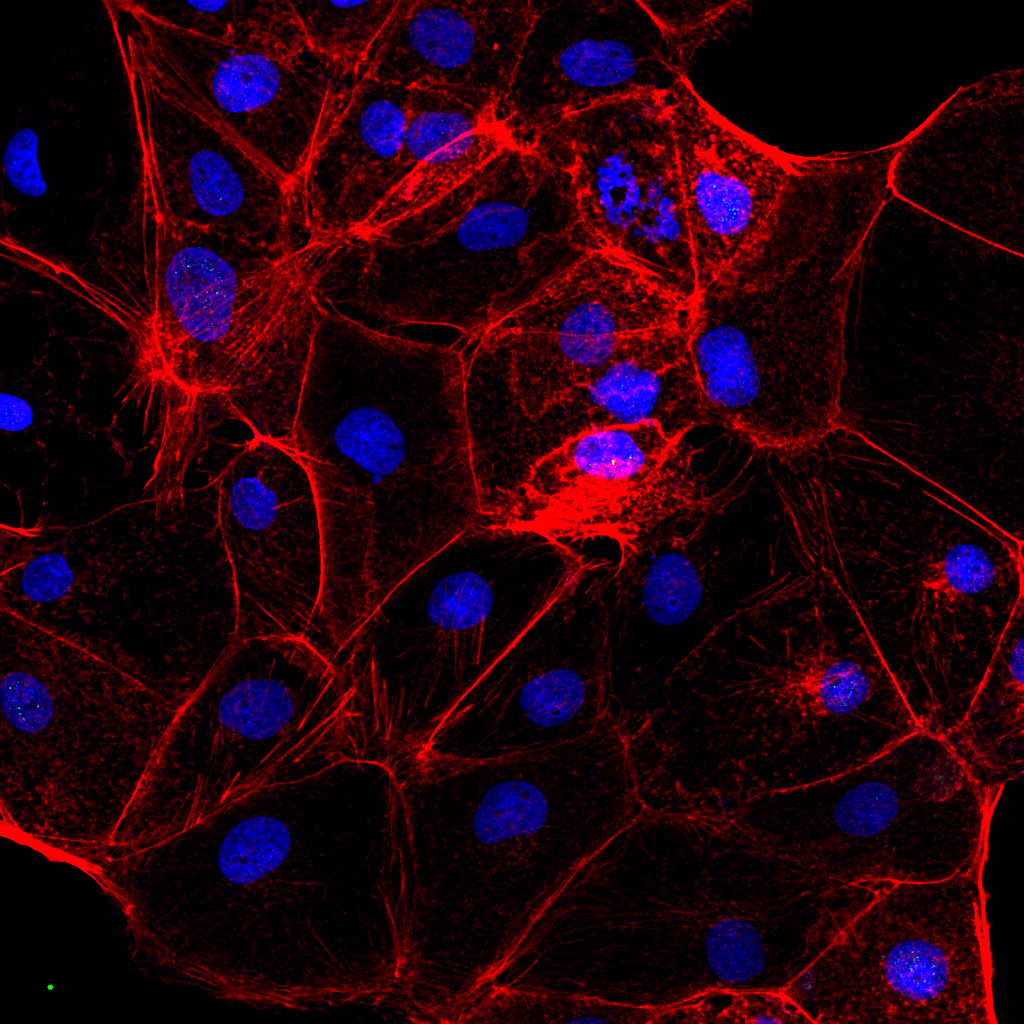

Supplement: Supplementary file 2 [file DataSheet1.ZIP › images/Figure 3/Figure 3 C d.tif]

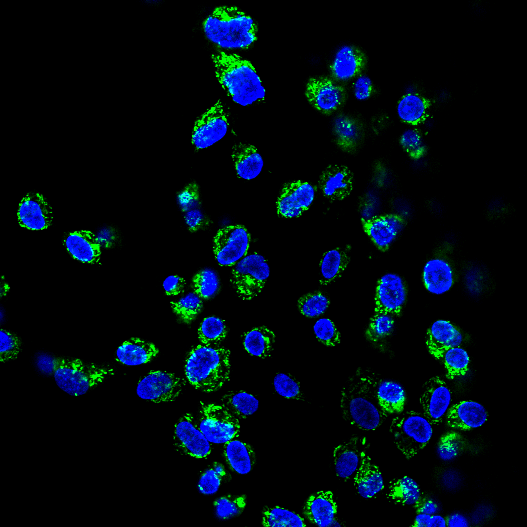

Supplement: Supplementary file 2 [file DataSheet1.ZIP › images/Figure 3/Figure 3 D a.tif]

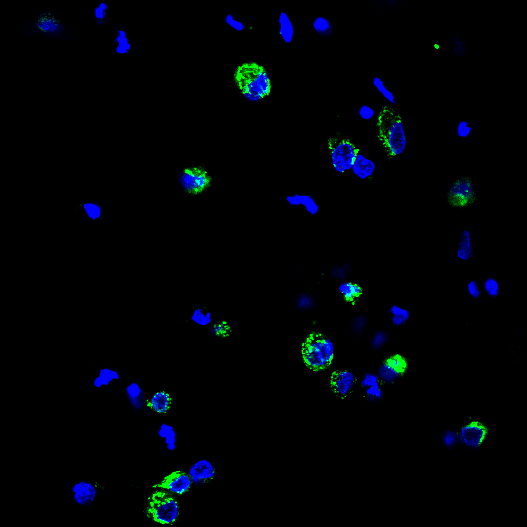

Supplement: Supplementary file 2 [file DataSheet1.ZIP › images/Figure 3/Figure 3 D b.tif]

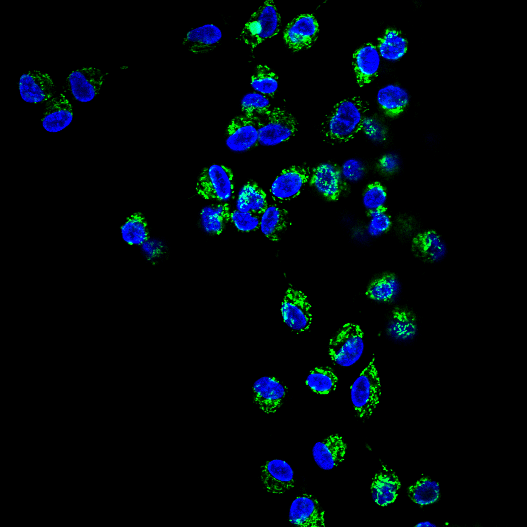

Supplement: Supplementary file 2 [file DataSheet1.ZIP › images/Figure 3/Figure 3 D c.tif]

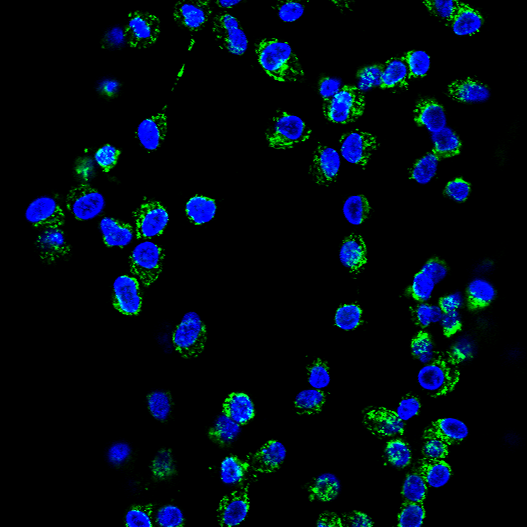

Supplement: Supplementary file 2 [file DataSheet1.ZIP › images/Figure 3/Figure 3 D d.tif]

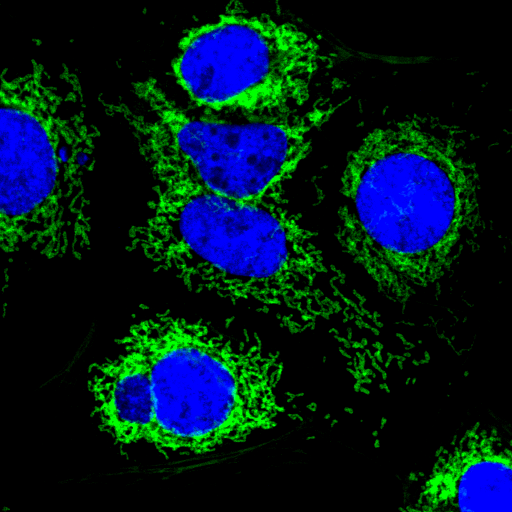

Supplement: Supplementary file 2 [file DataSheet1.ZIP › images/Figure 3/Figure 3 E a.tif]

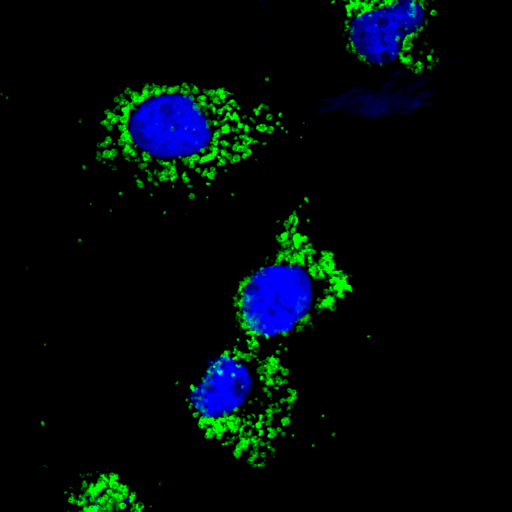

Supplement: Supplementary file 2 [file DataSheet1.ZIP › images/Figure 3/Figure 3 E b.tif]

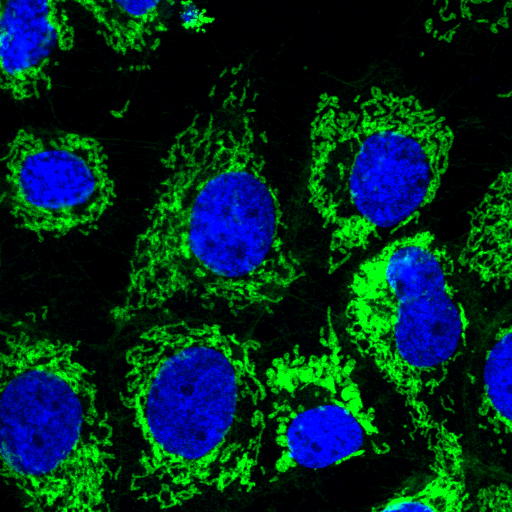

Supplement: Supplementary file 2 [file DataSheet1.ZIP › images/Figure 3/Figure 3 E c.tif]

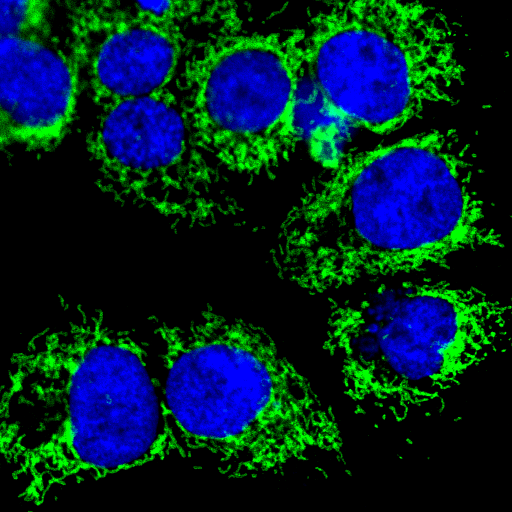

Supplement: Supplementary file 2 [file DataSheet1.ZIP › images/Figure 3/Figure 3 E d.tif]

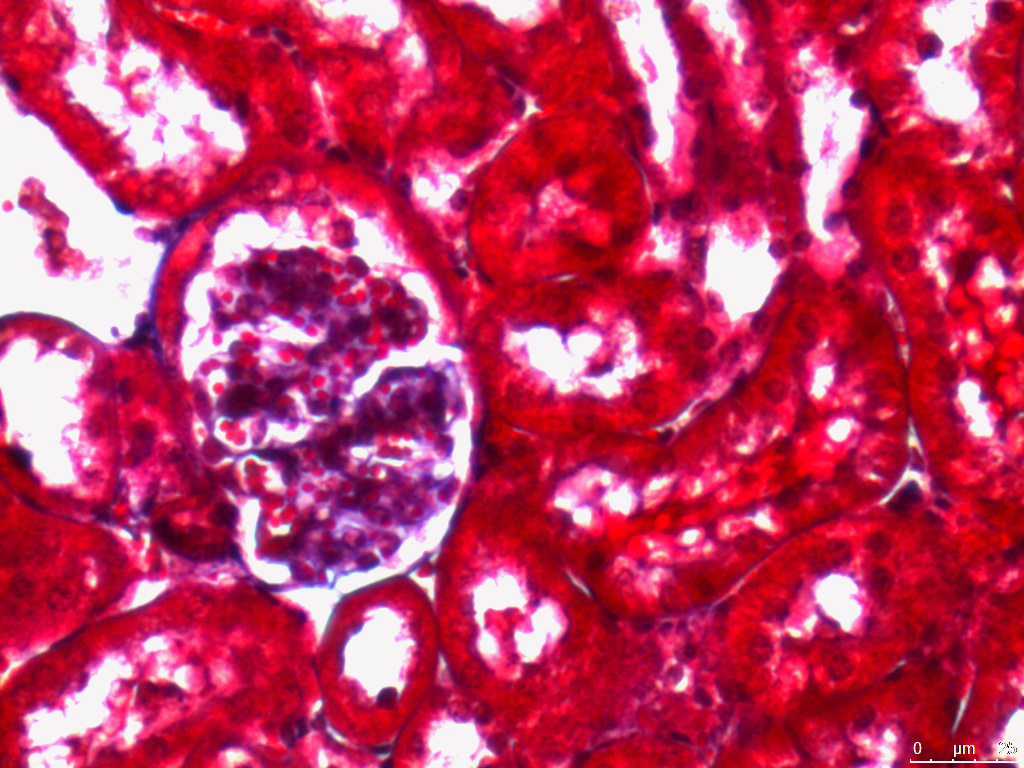

Supplement: Supplementary file 2 [file DataSheet1.ZIP › images/Figure 4/Figure 4 E a.jpg]

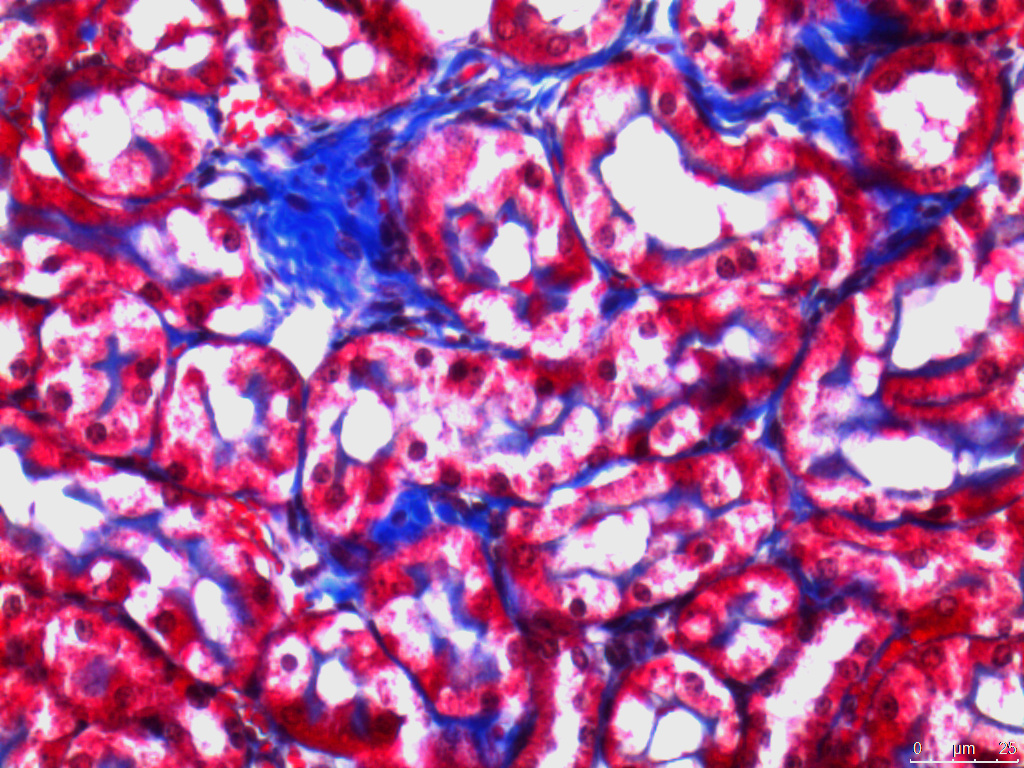

Supplement: Supplementary file 2 [file DataSheet1.ZIP › images/Figure 4/Figure 4 E b.jpg]

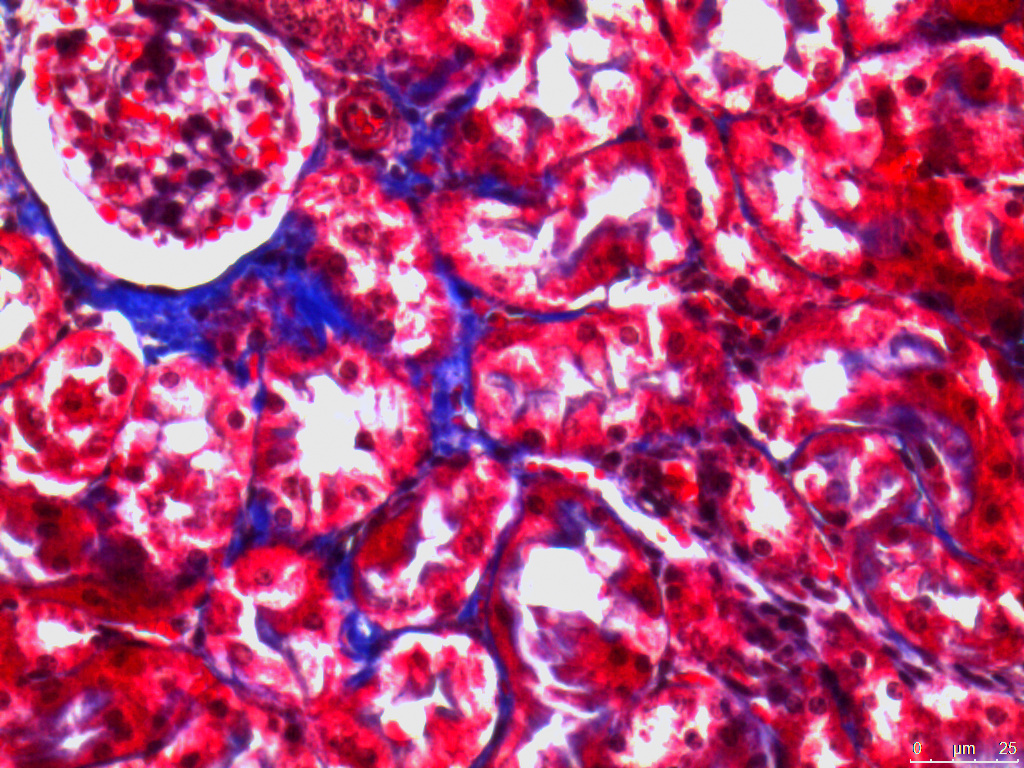

Supplement: Supplementary file 2 [file DataSheet1.ZIP › images/Figure 4/Figure 4 E c.jpg]

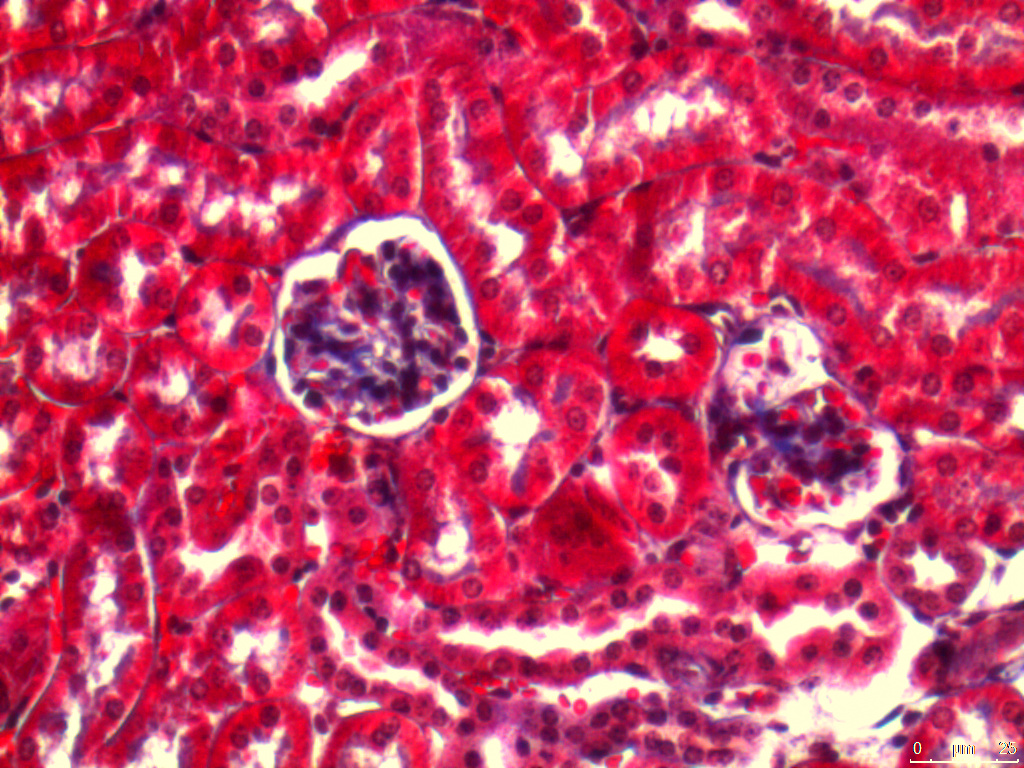

Supplement: Supplementary file 2 [file DataSheet1.ZIP › images/Figure 4/Figure 4 E d.jpg]

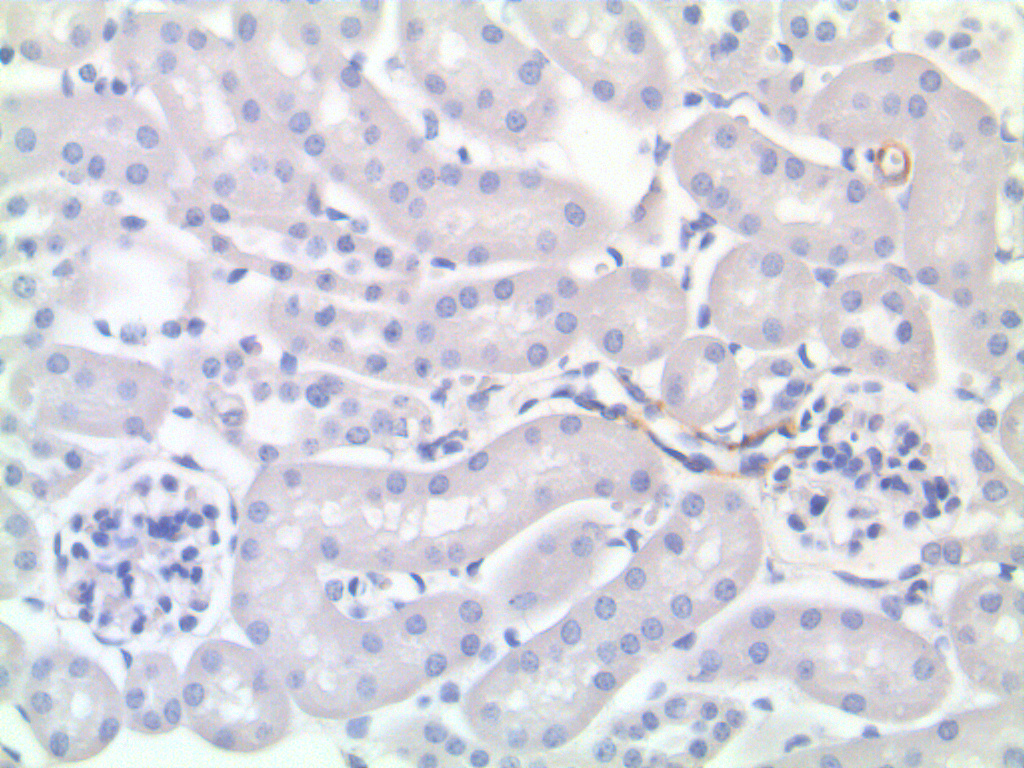

Supplement: Supplementary file 2 [file DataSheet1.ZIP › images/Figure 4/Figure 4 F a.jpg]

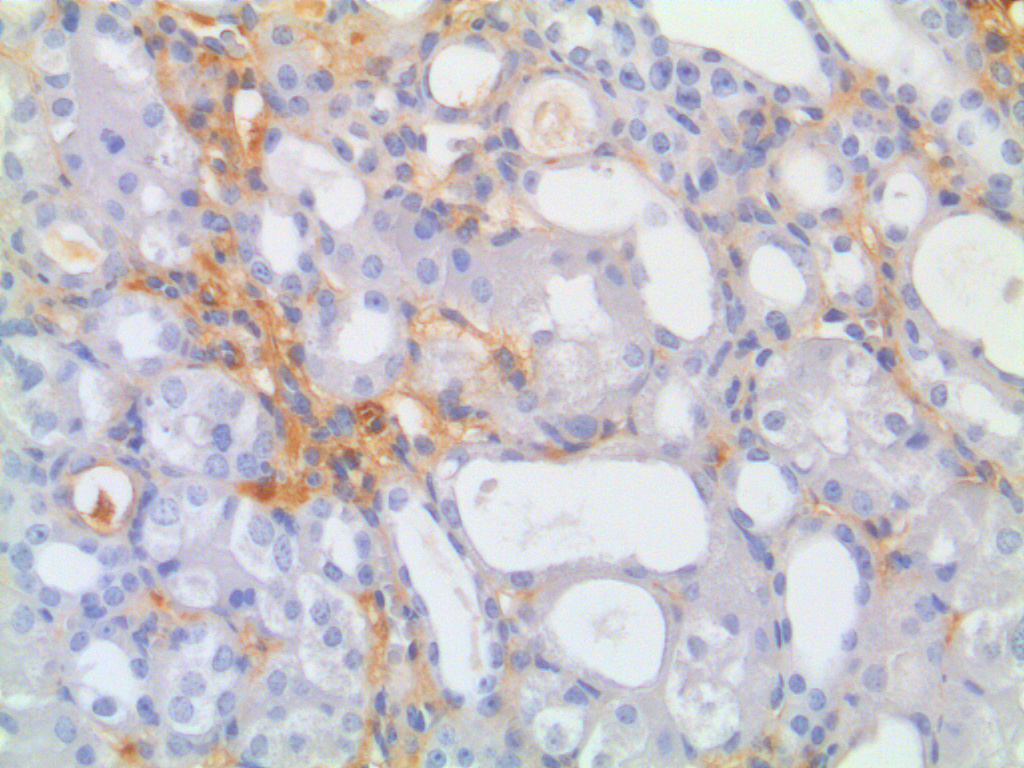

Supplement: Supplementary file 2 [file DataSheet1.ZIP › images/Figure 4/Figure 4 F b.jpg]

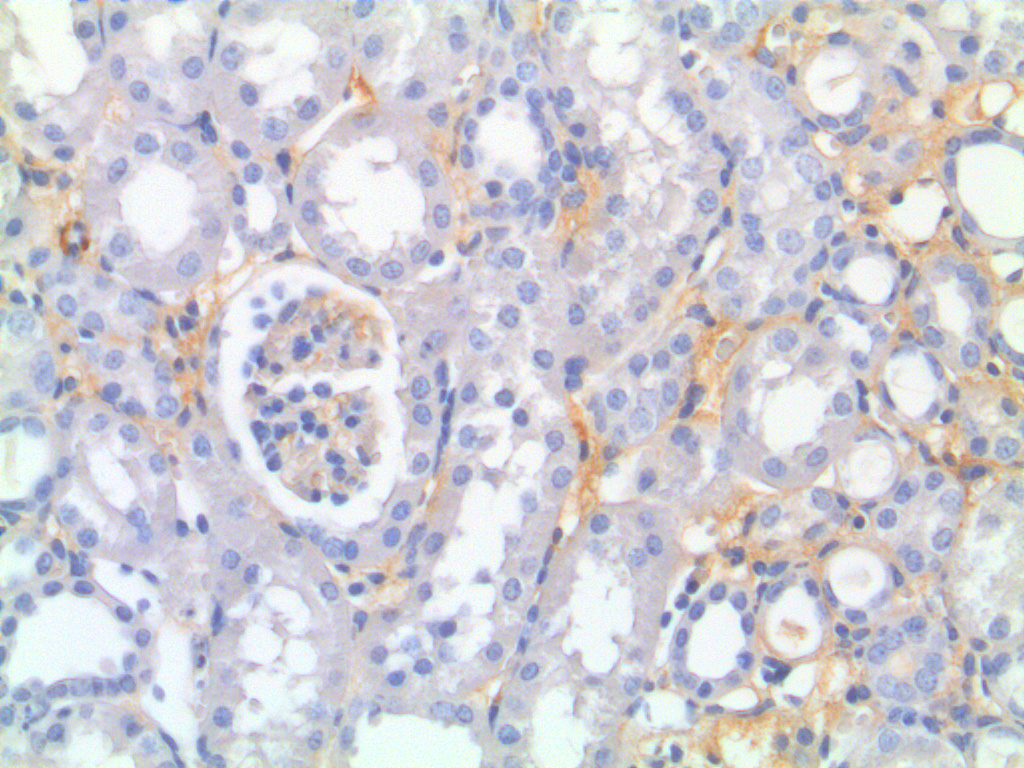

Supplement: Supplementary file 2 [file DataSheet1.ZIP › images/Figure 4/Figure 4 F c.jpg]

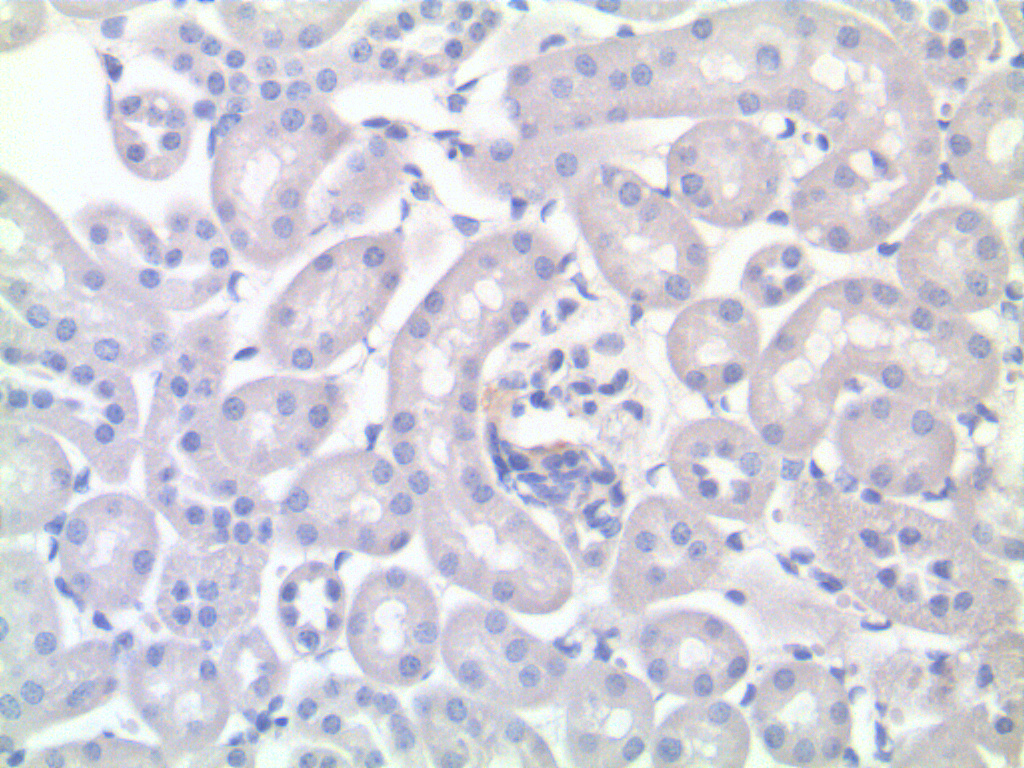

Supplement: Supplementary file 2 [file DataSheet1.ZIP › images/Figure 4/Figure 4 F d.jpg]

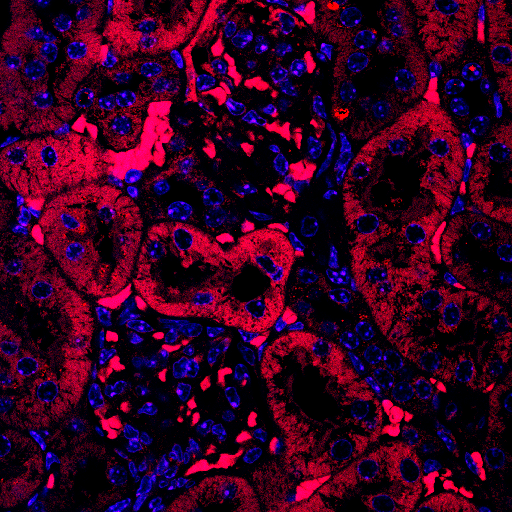

Supplement: Supplementary file 2 [file DataSheet1.ZIP › images/Figure 5/Figure 5 C a.tif]

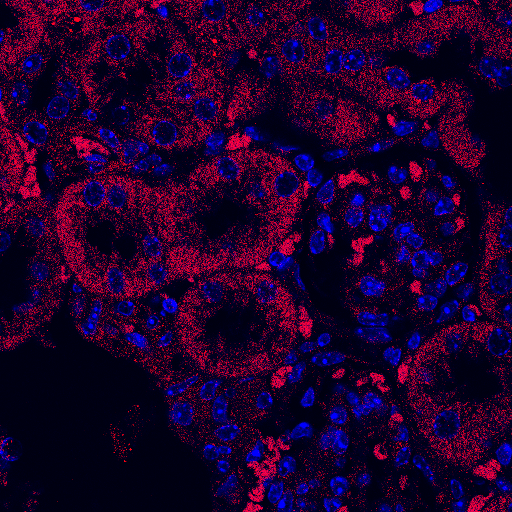

Supplement: Supplementary file 2 [file DataSheet1.ZIP › images/Figure 5/Figure 5 C b.tif]

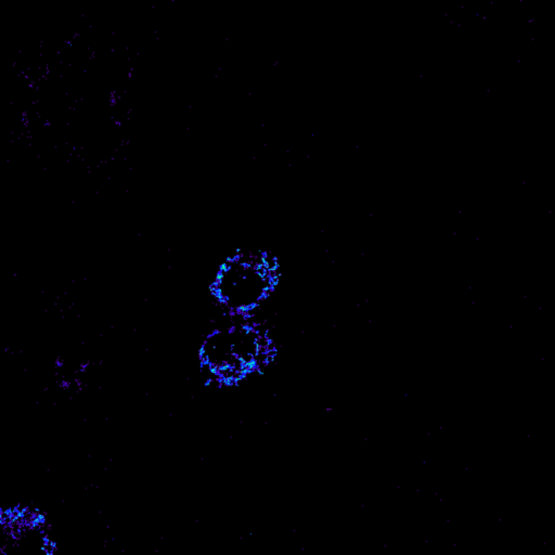

Supplement: Supplementary file 2 [file DataSheet1.ZIP › images/Figure 6/Figure 6 F a.tif]

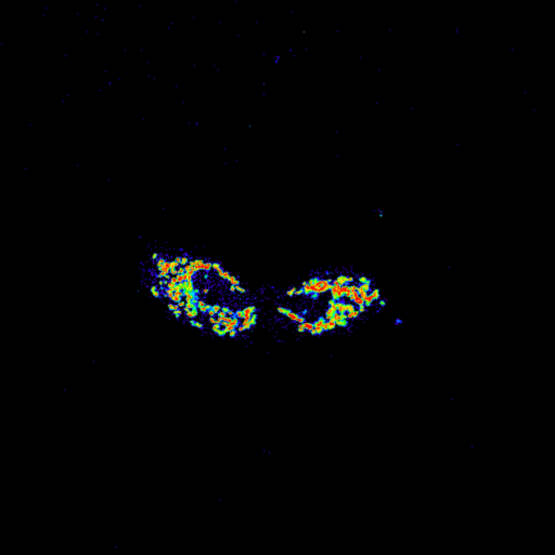

Supplement: Supplementary file 2 [file DataSheet1.ZIP › images/Figure 6/Figure 6 F b.tif]

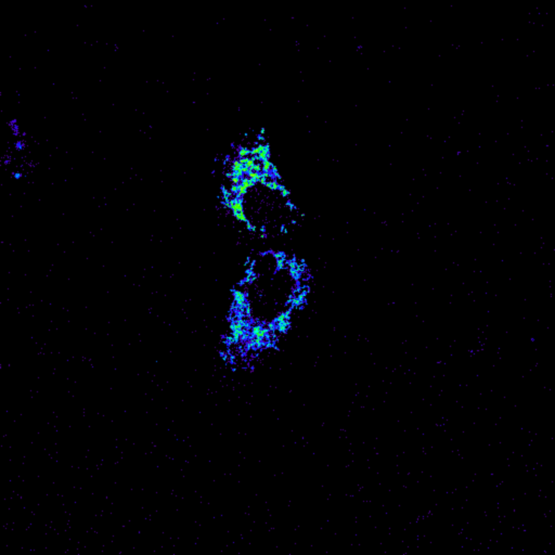

Supplement: Supplementary file 2 [file DataSheet1.ZIP › images/Figure 6/Figure 6 F c.tif]

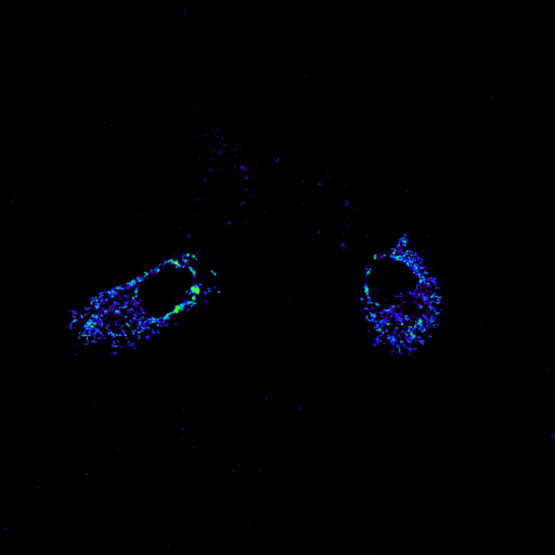

Supplement: Supplementary file 2 [file DataSheet1.ZIP › images/Figure 6/Figure 6 F d.tif]

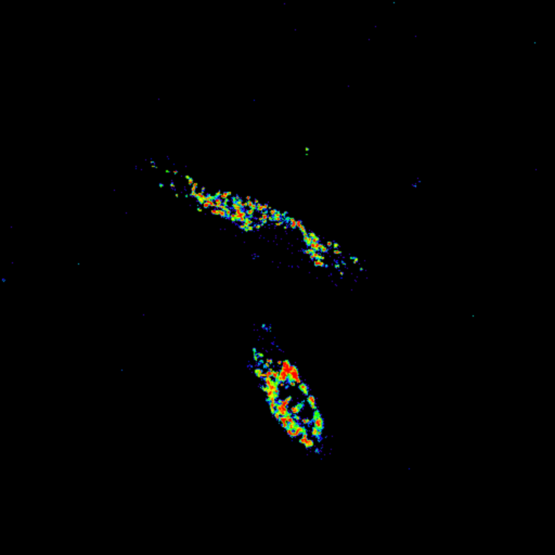

Supplement: Supplementary file 2 [file DataSheet1.ZIP › images/Figure 6/Figure 6 F e.tif]

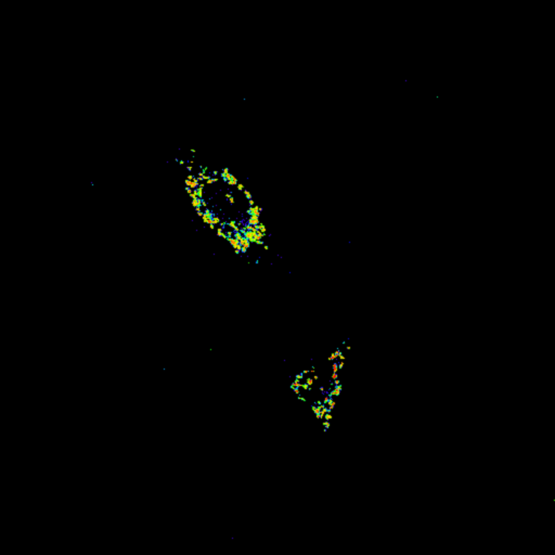

Supplement: Supplementary file 2 [file DataSheet1.ZIP › images/Figure 6/Figure 6 F f.tif]
