# Supplementary material for: Astragaloside IV alleviates 1-deoxysphinganine-induced mitochondrial dysfunction during the progression of chronic kidney disease through p62-Nrf2 antioxidant pathway
Source: Front Pharmacol. 2023 Mar 24;14:1092475. doi: 10.3389/fphar.2023.1092475 (PMC10079923; doi:10.3389/fphar.2023.1092475)
Supplement: Supplementary file 3 [file Image1.pdf]

Supplementary Information

Supplementary Figures

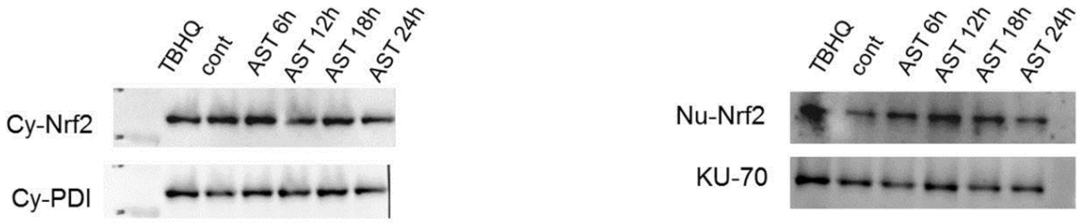

Figure S1 Representative WB images for Nrf2, PDI, and Ku-70 of HK-2 cells after exposure to the different treatment. 25μM TBHQ (tert-butylhydroquinone) was used as a positive control to induce nuclear translocation of Nrf2.
